# Placental cord drainage in the third stage of labor: Randomized clinical trial

**DOI:** 10.1371/journal.pone.0195650

**Published:** 2018-05-02

**Authors:** Fernanda Barros Vasconcelos, Leila Katz, Isabela Coutinho, Vanessa Laranjeiras Lins, Melania Maria de Amorim

**Affiliations:** Department of Obstetrics, Instituto de Medicina Integral Prof. Fernando Figueira, Recife, Pernambuco, Brazil; Public Library of Science, UNITED KINGDOM

## Abstract

**Methods:**

An open randomized clinical trial was developed at *Instituto de Medicina Integral Prof*. *Fernando Figueira* (IMIP) in Recife and at *Petronila Campos* Municipal Hospital in São Lourenço da Mata, both in Pernambuco, northeastern Brazil, including 226 low-risk pregnant women bearing a single, full-term, live fetus after delayed cord clamping, 113 randomized to placental cord drainage and 113 to a control group not submitted to this procedure. Women incapable of understanding the study objectives and those who went on to have an instrumental or cesarean delivery were excluded.

**Results:**

Duration of the third stage of labor did not differ between the two groups (14.2±12.9 versus 13.7±12.1 minutes (mean ± SD), p = 0.66). Likewise, there was no significant difference in mean blood loss (248±254 versus 208±187ml, p = 0.39) or in postpartum hematocrit levels (32.3±4.06 versus 32.8±4.25mg/dl, p = 0.21). Furthermore, no differences were found between the groups for any of the secondary outcomes (postpartum hemorrhage >500 or >1000ml, therapeutic use of oxytocin, third stage >30 or 60 minutes, digital evacuation of the uterus or curettage, symptoms of postpartum anemia and maternal satisfaction).

**Conclusion:**

Placental cord drainage had no effect in reducing duration or blood loss during the third stage of labor.

**Clinical trials registration:**

ClinicalTrials.gov: www.clinicaltrial.gov, NCT01655576.

## Introduction

Postpartum hemorrhage accounts for 127,000 deaths annually worldwide [1] and its incidence is increasing in developed nations [2–9]. It is the major cause of maternal mortality globally [10–16] and the second cause of maternal mortality in Brazil [8]. The active management during the third stage of labor is recommended as a preventive strategy [2].

Active management consists of measures to reduce the duration of the third stage of labor and the blood loss that occurs during this stage [17]. Uterotonics and immediate umbilical cord clamping are techniques that have been proposed [2]. Uterine massage is no longer recommended for the prevention of postpartum hemorrhage and controlled cord traction has already been shown to be ineffective in vaginal deliveries [2]. Current recommendations state that cord ligation should be postponed in view of the known benefits to the neonate [2].

A different strategy for accelerating uterine emptying is placental cord drainage, which involves clamping and cutting the umbilical cord following delivery of the baby and then immediately unclamping the maternal end of the cord, allowing the blood to flow freely into a container until successful uterine emptying [18].

The few randomized clinical trials conducted to evaluate placental cord drainage have shown a significant reduction in the duration of the third stage of labor following drainage [19,20]. A Cochrane review also revealed a reduction in the duration of the third stage of labor with placental cord drainage [18]. Nevertheless, placental cord drainage is still not used routinely in clinical practice.

The purpose of the present study was, therefore, to determine the effectiveness of placental cord drainage in the third stage of labor.

## Methods

An open, randomized clinical trial was conducted to compare women submitted to placental cord drainage in the third stage of labor with a control group. The study was developed at *Instituto de Medicina Integral Prof*. *Fernando Figueira* (IMIP) in Recife and at *Petronila Campos* Municipal Hospital in São Lourenço da Mata, both in Pernambuco, northeastern Brazil between February and May 2013. The study was approved by IMIP’s internal review board under reference number 03139112.2.0000.5201. All participants voluntarily agreed to participate in the study and signed an informed consent form.

Sample size was calculated using the OpenEpi software program, version 2.3 (Atlanta, Georgia, USA), based on a previous randomized clinical trial that showed a reduction in the duration of the third stage of labor with the use of placental cord drainage (5.1±2.4 versus 7.0±6.1minutes; mean/standard deviation)[19]. Considering a two-sided t test, significance level of 5% and a power of 80%, 188 women would be required, this number was increased to 226 to compensate for losses.

The participants were low-risk pregnant women bearing a single, full-term live fetus. Women who went on to have a Cesarean section or instrumental delivery were excluded.

Randomization was performed according to a computer-generated list of numbers produced using the Random Allocation software program, version 1.0 (Isfahan, Iran). Sealed envelopes numbered from 1 to 226 were prepared by an assistant who was not involved in the data collection.

Following spontaneous vaginal delivery, after delayed cord clamping, a numbered envelope was opened to reveal the woman’s group assignment. Immediately after the birth, all women received two ampoules of 10IU oxytocin intramuscularly as part of routine care provided [2].

Placental cord drainage consisted of clamping and cutting the umbilical cord following delivery of the infant and then immediately unclamping the maternal end of the cord, allowing the blood from the placenta to drain freely into a container [21], which was not the one used to measure blood loss. In the control group, the cord remained clamped until delivery of the placenta. The procedure in both groups were conducted only after delayed cord clamping.

The primary outcomes were: duration of the third stage of labor (minutes), volume of blood loss in the first hour postpartum (ml) and postpartum hematocrit. The amount of blood lost was calculated from the blood drained into a plastic bag, placed under the woman’s buttocks immediately following delivery and drained into an appropriate stainless-steel recipient until delivery of the placenta. No gauze pads or compresses were used at this time. Blood samples were taken prior to and 24–48 hours after delivery to measure hematocrit and hemoglobin levels.

The following secondary outcomes were analyzed: postpartum hemorrhage (>500 ml) or severe postpartum hemorrhage (>1000 ml) in the first hour, need for blood transfusion, postpartum abdominal pain, need for oxytocin in the first hour and in the first 24 hours postpartum, third stage of labor >30 or >60 minutes, digital evacuation of the uterus, manual removal of placenta, uterine curettage, symptoms of anemia in the 48 hours postpartum and maternal satisfaction with the management of the third stage of labor.

Maternal satisfaction regarding how the third stage of labor was managed was evaluated by applying a satisfaction scale ranging from 1 to 5 (very satisfied, satisfied, not very satisfied, unsatisfied and very unsatisfied) approximately 24–48 hours following delivery [22]. For the purposes of analysis, all women who answered *satisfied* or *very satisfied* were considered to have been satisfied with the procedure.

Data analysis was performed by a blinded statistician using Epi-Info software program, version 7 (CDC, Atlanta, GA, USA). Analysis was planned to be conducted on an intention-to-treat basis. During analysis, the groups remained identified as A or B, with randomization only being revealed when analysis was complete.

Tables were constructed to evaluate the association between the independent or predictor variable and the dependent variables. For numerical variables, Student’s t test or Mann-Whitney test was used, if appropriate. For categorical variables, Pearson’s chi-square test or Fisher’s exact test was used as appropriate. If data was nonparametric or not normally distributed, Mann-Whitney was used, and indicated in tables. All p-values were two-tailed. Risk ratios (RR) were calculated as measures of relative risk, together with their respective 95% confidence intervals (95%CI).

## Results

**Fig 1:**
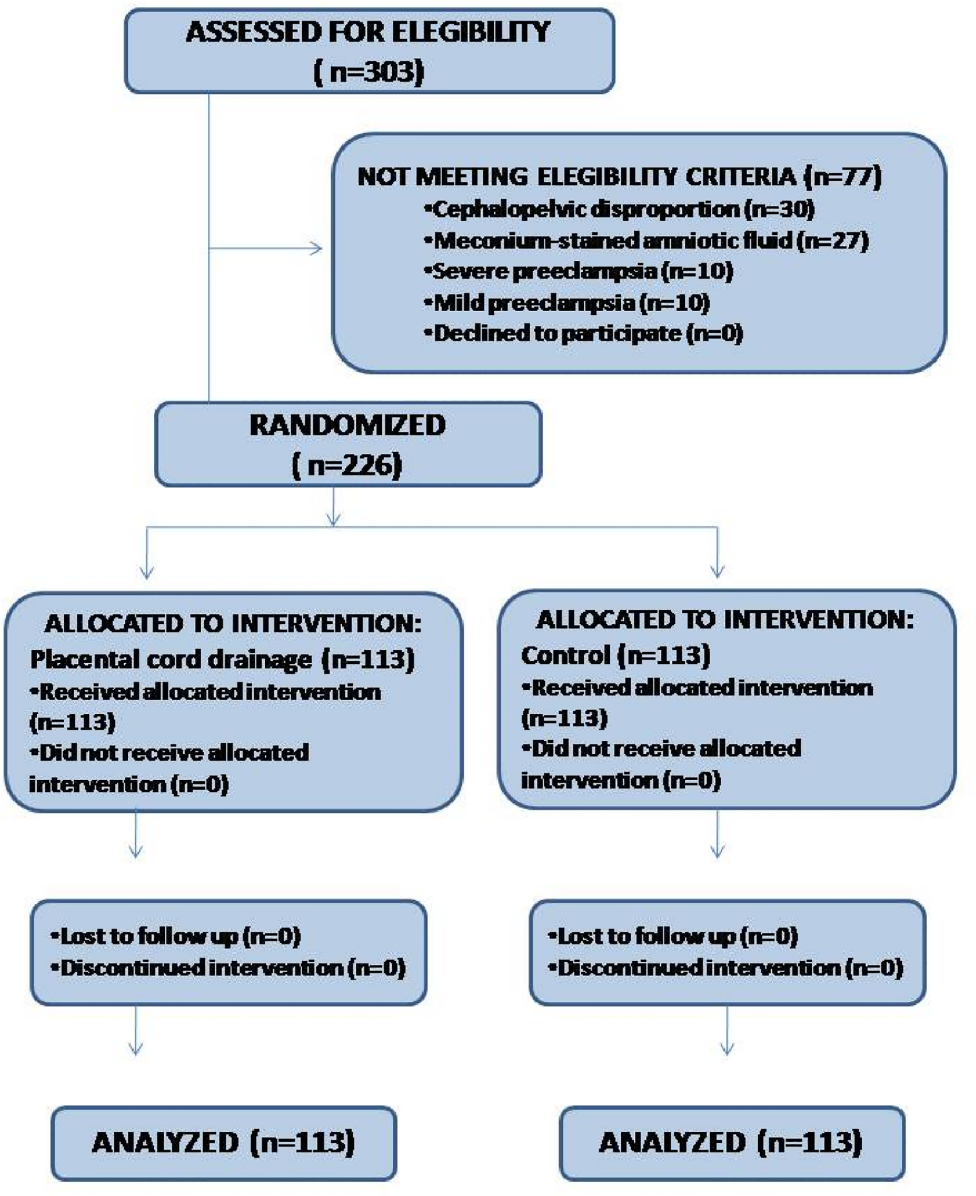
Study flowchart.

During the study period, 251 women were approached and 226 randomized, 113 to the placental cord drainage and 113 to the control group (Fig 1).

The groups were similar with respect to the sociodemographic and obstetric characteristics and pre-delivery hematocrit levels, the only difference being in mean age, which was lower in the intervention group (22.7 versus 24.5 years, p = 0.02). Analysis of the percentage of women ≥35 years of age also found no difference between the groups (5.3% versus 9.7%, p = 0.20) (Table 1).

**Table 1:** Baseline characteristics of the study participants.

Characteristics	Placental cord drainage		Control		p-value
Age (mean/SD)	22.7	6.3	24.5	6.3	0.02*
Age ≥ 35 years (n/%)	6	5.3	11	9.7	0.20
Schooling (mean/SD)	9.6	2.8	9.9	2.8	0.25*
Pregnancies (median/IQR)	1	1–2	2	1–3	0.12*
Parity (median/IQR)	0	0–1	1	0–1	0.08*
Black/brown-skinned (n/%)	75	66.4	86	76.1	0.10
Body mass index (mean/SD)	26.4	3.8	27.1	3.9	0.10*
Having a partner (n/%)	95	84.1	93	82.3	0.72
Delivery in vertical position (n/%)	107	94.7	107	94.7	1.00
Episiotomy performed (n/%)	1	0.9	0	0	0.31
Spontaneous tearing (n/%)	88	77.9	92	81.4	0.50
Pre-delivery hematocrit (mean/SD)	35.7	3.07	36.0	3.62	0.37*

IQR: interquartile range; SD: standard deviation

* Mann-Whitney

There was no statistically significant difference in the mean duration of the third stage of labor between the groups: 14.2±12.9 versus 13.7±12.1minutes (mean/standard deviation; Mann-Whitney) for the intervention and control groups, respectively (p = 0.66). Likewise, no statistically significant difference was found in mean blood loss (248±2.54 versus 208±187ml; mean/standard deviation; p = 0.39; Mann-Whitney;) or in postpartum hematocrit (32.2±4.06 versus 32.8±4.25mg/dL; p = 0.21; mean/standard deviation; Mann-Whitney) (Table 2).

**Table 2:** Primary and secondary maternal outcomes.

Primary maternal outcomes	Placental cord drainage		Control group		p-value*
	mean	SD	mean	SD	
Duration of the third stage of labor (minutes)	14.2	12.9	13.7	12.1	0.66
Blood loss (ml)	248	254	208	187	0.39
Hematocrit 24–48 h postpartum	32.2	4.06	32.8	4.25	0.21

SD: standard deviation

*Mann-Whitney

The frequency of secondary outcomes was also similar in both groups. Postpartum hemorrhage >500 ml was registered in 10.6% versus 8% of cases (RR = 1.3; 95%CI: 0.58–3.09) in the intervention and control groups, respectively. Postpartum hemorrhage >1000 ml occurred in 3.5% versus 2.7% (RR = 1.3%; 95%CI: 0.30–5.82) and hemoglobin <8g/dl in the first 24–48 hours postpartum in 3.5% versus 0.9% (RR = 4.0; 95%CI: 0.45–35.23). Most of the women complained of postpartum abdominal pain: 51.6% versus 48.4% (p = 0.68); however, few required uterotonics in the first hour or in the first 24 hours postpartum (3.5% versus 0.9%; p = 0.18 and 1.8% versus 0.9%; p = 0.50, for the intervention and control groups, respectively) (Table 3).

**Table 3:** Secondary maternal outcomes.

Secondary maternal outcomes	Placental cord drainage		Control Group		RR	95%CI	p-value
	n	%	n	%			
Postpartum hemorrhage > 500 ml	12	10.6	9	8.0	1.3	0.58–3.09	0.49
Postpartum hemorrhage >1000 ml	4	3.5	3	2.7	1.3	0.30–5.82	0.50^a^
Maternal hemoglobin <8g/dl 24–48 h postpartum	4	3.5	1	0.9	4.0	0.45–35.23	0.18^a^
Postpartum abdominal pain	47	51.6	44	48.4	1.06	0.77–1.46	0.68
Oxytocin in the first hour postpartum	4	3.5	1	0.9	4.0	0.45–35.23	0.18^a^
Oxytocin in the first 24h postpartum	2	1.8	1	0.9	2.0	018–21.74	0.50^a^
Third stage of labor > 30 minutes	9	8.0	10	8.8	0.9	0.38–2.13	0.81
Third stage of labor > 60 minutes	1	0.9	0	0			0.50^a^
Digital evacuation of the uterus	6	5.3	4	3.5	1.5	0.43–5.17	1.37^a^
Manual removal of placenta	4	3.5	1	0.9	4.0	0.48–35.2	0.18^a^
Curettage	3	2.7	3	2.7	1.0	0.20–4.84	0.65
Symptoms of anemia up to 48 h postpartum	15	13.4	12	10.7	1.2	0.61–2.54	0.53^a^
Maternal satisfaction good or very good	50	44.2	45	39.8	1.1	0.81–1.51	0.50

RR: relative risk, CI: confidence interval,

^a^ Fisher’s exact

Few cases of third stage of labor >30 or >60 minutes were recorded in the intervention and control groups: 8.0% versus 8.8% (p = 0.81) and 0.9% versus 0% (p = 0.50), respectively. The frequency of digital evacuation of the uterus, manual removal of the placenta and curettage were similar in both groups. Overall, 13.4% of women in the intervention group and 10.7% in the control group showed symptoms of anemia in the first 48 hours postpartum (p = 0.53) and most women were satisfied with how the third stage of labor was managed (44.2% versus 39.8% in the intervention and control groups, respectively; p = 0.50) (Table 3).

## Discussion

A Cochrane review evaluated the effects of placental cord drainage in third stage of labor. Three clinical trials with 1257 low-risk women were evaluated; however, in addition to placental cord drainage, all trials included other procedures such as immediate umbilical cord ligation and controlled cord traction as part of the management of the third stage of labor. Furthermore, postpartum use of uterotonics varied between studies. Placental cord drainage was found to reduce the third stage of labor by around three minutes, with a slight reduction in blood loss [18]. The authors of the meta-analysis warned, however, that the results should be interpreted with caution, since the reduction in the duration of the third stage of labor was small and the studies were heterogenous.

Previous studies suggested that placental cord drainage reduces the duration of the third stage of labor and blood loss [18,19,23,24,25]. In a randomized clinical trial, including 49 women in the intervention group and 50 in a control group, a significant reduction in the duration of third stage of labor was found following drainage (5.1±2.4 versus 7.0±6.1minutes)[19]. Nevertheless, in the present study, no difference was found (approximately 14 minutes in both groups; p = 0.66).

In this study, the duration of the third stage of labor was longer in both groups compared to the previous study. Since management of third stage of labor in that study included maneuvers such as immediate clamping, controlled cord traction and uterine massage, which are no longer recommended, it is possible that these maneuvers may have shortened the time elapsed. In addition to the fact that the study sample was small, the difference in the duration of the third stage was of around two minutes, which, although statistically significant, is not clinically relevant [19]. The present study, which was conducted in accordance with current WHO recommendations [2] and with a larger sample size, failed to confirm the previous findings.

Another randomized clinical trial compared 239 women submitted to placental cord drainage plus controlled cord traction and 238 women submitted to expectant management. The median duration of the third stage of labor was significantly shorter in the intervention group (8 versus 15 minutes; p<0.001) [23]. That study did not evaluate placental cord drainage alone but, rather, in association with controlled cord traction, a procedure not routinely recommended by WHO [2]. It is difficult to estimate the value of each one of these procedures alone, hence impossible to compare those results with other studies.

Controlled cord traction, despite guidelines recommending otherwise [2] was used in routinely in all patients of both groups included in a randomized clinical trial including 200 patients randomized to placental drainage in addition to active management or active management only. The authors found difference in third period duration with placental drainage (3,5 x 5,0 minutes)[25]. Not surprisingly, duration was shorter than reported in previous studies, and the one found in the present study. It is also important to point out that, although statistically different, this difference in time is not clinically important. The same result was found in a study [26], with a larger sample size, randomized 242 women to unclamping the cord after cutting and 243 to a control group were the cord remained clamped. The duration of third stage of labor was significantly shorter with placental cord drainage (3.5 versus 7.7 minutes, p<0.001). As remarked before, although third stage was about four minutes shorter, it is probable that a four minutes reduction is not clinically relevant.

Considering blood loss volume, previous results vary. One study describes 200 women randomized to placental drainage versus maintaining the cord clamped [27]. The volume of blood loss was smaller in the first group (175 versus 252ml). Controlled cord traction and methylergometrine were used in both groups. Different management of the third period of labor probably explains this difference. It is also important to point out that the volume difference found in the first study is not clinically significant. In the second study [26] with a larger sample size (485 women), blood loss was one of the primary outcomes analyzed. The authors found that blood loss was lower with placental cord drainage (207 versus 277ml, p<0.001). Again, although statistically significant it is unlikely that losing 70ml less blood volume is clinically relevant.

In another study, blood loss was evaluated based on postpartum hemoglobin levels, mean reduction in hemoglobin and percentage of cases in which hemoglobin decreased >3g/dl. Although the mean reduction in hemoglobin was greater in women not submitted to placental cord drainage, mean postpartum hemoglobin levels and frequency of women with postpartum hemoglobin <10g/dl were similar in both groups [23]. This difference did not persist, however, when only those women without episiotomy and with an intact perineum were evaluated. It is possible that the different types of management adopted may have affected the findings.

Finally, in the study that included 200 patients randomized to placental drainage in addition to active management or active management only, results showed that blood loss was significantly lower in the placental drainage group. Another observation was that the change in maternal hemoglobin before and after labor and the percentage of patient that experienced postpartum hemorrhage was lower with placental drainage [25]. About this study, some issues need to be pointed out, as the routine use of controlled cord traction and the lack of information about the moment of cord clamping.

The present study also evaluated postpartum hematocrit and frequency of women with hemoglobin <8 g/dl; however, no statistically significant differences were found. In relation to frequency of postpartum hemorrhage, the findings from the present study corroborate those of other authors who reported no differences with the use of this procedure [19,20,23]. There may be small differences in blood loss; however, it would take a larger sample size to detect differences in significant postpartum hemorrhage (>500 or >1000 ml). Even with a larger sample size such as that included in the Cochrane meta-analysis, although a small reduction in the volume of blood loss was found, there was no difference in the risk of postpartum hemorrhage or in the need for a blood transfusion [18].

A recent meta-analysis including 9 studies and 2653 patients found that the duration of third stage of labor is shortened (2,28 minutes) although blood loss was the same [28]. A new finding was the reduction of 3% in postpartum hemorrhage. This finding is surprising since there is no reduction in blood loss. Since there studies were very heterogenous, caution is needed before drawing definitive conclusions. The meta-analysis suggests that placental drainage is a simple and noninvasive procedure that seems to add to the management of patients, but more studies are still necessary to clarify its importance.

Other outcomes such as need to remove the placenta, postpartum abdominal pain, postpartum oxytocin use and symptoms of postpartum anemia were also analyzed in the present study, with no differences being found between the groups. Nevertheless, comparison with the results of other studies was impossible since, none of the included studies had described these variables.

One explanation for the present findings that placental cord drainage has no effect either on the duration of the third stage of labor or on blood loss may be that the sample consisted of pregnant women at little risk of developing hemorrhage. Furthermore, all participants received prophylactic oxytocin for preventing postpartum hemorrhage, whereas in the other studies the management of third stage of labor varied, possibly affecting the results. It should also be emphasized that this study was conducted in settings that follow a policy of avoiding interventions such as episiotomy and prepartum oxytocin, which are factors that increase the risk of hemorrhage [1], thus possibly explaining the absence of any effect of placental cord drainage in this population.

One of the strong points of the study is the evaluation of the volume of blood loss, thus eliminating observer bias. Furthermore, analyzing women in a setting in which interventions are few, reduces the possibility that practices such as episiotomy, for example, could affect the results. Another strong point of the study is that placental cord drainage was the only technique analyzed, i.e. the only difference in the procedures applied to the groups.

On the other hand, one of the limitations of the study was that the women included were at a low risk for the event. Therefore, larger sample sizes, as well as the inclusion of high-risk women and/or use of different practices during delivery may be necessary to identify the effect of this procedure.

## Conclusions

In the present study, placental cord drainage had no effect on the duration of the third stage of labor or on postpartum blood loss. Although initial studies have been encouraging, the present study failed to confirm the efficacy of this practice. We recommend further evaluation of placental cord drainage in future studies before its use can be established in routine clinical practice. On the other hand, since placental cord drainage is harmless, it may be performed whenever considered necessary by the professional providing care at delivery.

## Supporting information

S1 File Methods. (PDF)

S2 File Methods (english). (PDF)

S3 File CONSORT checklist. (DOC)

S4 File Database. (MDB)
