# Supplementary material for: Placental cord drainage in the third stage of labor: Randomized clinical trial
Source: PLoS One. 2018 May 2;13(5):e0195650. doi: 10.1371/journal.pone.0195650 (PMC5931461; doi:10.1371/journal.pone.0195650)
Supplement: S1 File — (PDF) [file pone.0195650.s001.pdf]

INSTITUTO DE MEDICINA INTEGRAL PROF. FERNANDOFIGUEIRA-IMIP
PROGRAMA DE MESTRADO EM SAÚDE MATERNO INFANTIL

EFETIVIDADE DA DRENAGEM PLACENTÁRIA NO TERCEIRO PERÍODO DO PARTO: ENSAIO CLÍNICO RANDOMIZADO

I. INTRODUÇÃO

O terceiro período do parto é compreendido entre o nascimento do neonato até a expulsão da placenta[1] , é o período que há maior suscetibilidade para a hemorragia pós-parto (HPP). Quando se adota conduta ativa no terceiro período, ele é diagnosticado como prolongado se não se completar em 30 minutos, já com conduta fisiológica ou expectante é diagnosticado como prolongado se não se completar em 60 minutos[2]. A contração e retração do músculo uterino que provocam a separação da placenta comprimem fortemente os vasos sanguíneos, controlando, desta forma, a hemorragia. Isso é possível devido à presença das fibras musculares oblíquas no segmento uterino superior[3]. Falha ou atraso na contração uterina após o parto pode levar à hemorragia[3].

Hemorragia pós-parto (HPP) representa a principal causa de mortalidade materna em todo o mundo[4] e é uma importante causa de morbidade nos países desenvolvidos e em desenvolvimento[5]. O diagnóstico dessa entidade é clínico[3] e para sua prevenção é recomendada conduta ativa no terceiro período do parto[6].

A definição mais amplamente aceita de HPP corresponde a perda de 500 ml de sangue ou mais, pelo trato genital, após o parto[7], sendo considerada grave quando a perda for acima de 1000 ml independente do tipo de parto[8]. A HPP pode ser primária ou secundária, em relação ao momento do evento. A primária é a hemorragia excessiva que ocorre nas 24 horas que seguem ao parto, enquanto a secundária, é o sangramento excessivo 24 horas após o nascimento até seis semanas após o parto[4,7].

Apesar do conceito clássico de HPP, ainda existem controvérsias. Nos Estados Unidos e Canadá, por exemplo, uma perda de sangue de 500 mL para um parto vaginal e 1.000 mL para um parto cesariana[9]. Uma outra definição é usada na Austrália, que considera uma perda de sangue de 500 mL para um parto vaginal e 750 mL para uma cesariana[10].

Vale salientar que o limite de 500 ml pode ser visto como um sinal de alerta para reconhecer que a perda de sangue até 1000 ml nas mulheres saudáveis pode ainda ser considerada fisiológica, evitando a progressão para quadros mais graves que implicam na realização de intervenções mais complexas como hemotransfusão[11].

Em países de baixa renda, onde a prevalência de anemia grave é alta, a perda 500 ml de sangue pode ser prejudicial para muitas mulheres. No entanto, mesmo em países desenvolvidos, o risco de HPP não deve ser subestimado para qualquer nascimento[11], visto que, apesar de ser mais comum em países em desenvolvimento, tem-se observado uma tendência crescente na incidência de HPP nos países desenvolvidos[12,13].

A hemorragia como causa de morte materna representa um indicador de saúde da população feminina. A morte materna como resultado de hemorragia é considerada uma perda evitável, denotando dessa forma, a falta de assistência adequada ao parto e ao pós-parto imediato, ocorrendo mais comumente em mulheres pobres e com maior paridade[12,14].

A HPP provoca 127 mil mortes anualmente em todo mundo e sua incidência vem aumentando em países desenvolvidos[6]. Um estudo realizado na Nigéria, observou que a morbidade mais comum foi a hemorragia pós-parto (35,4%)[15]. No Brasil, a hemorragia está entre as três primeiras causas de morbimortalidade materna. Um estudo realizado no Recife mostra que a hemorragia (16%) é segunda causa mais freqüente, onde a grande maioria dessas mortes poderia ter sido evitada, sobretudo pela assistência[16].

As principais causas de HPP são atonia uterina, retenção placentária, lacerações, hematomas, rotura uterina e coagulopatias[12] . Essa morbidade é mais comum nas multíparas, em mulheres com uma cesárea anterior, com diagnóstico de placenta prévia, submetidas à indução do parto, nas que apresentam trauma uterino ou cervical no momento do parto, na idade gestacional menor que 32 semanas e peso ao nascer maior

ou igual a 4500 g[17], Índice de Massa Corpórea (IMC) maior que 40[18], fase ativa do trabalho de parto prolongada[19], terceiro período do parto prolongado[18] e a utilização de intervenções obstétricas[20].

Independentemente das definições utilizadas, as estimativas visuais de perda sanguínea são frequentemente imprecisas[11], e as análises usando a perda de sangue demonstram que muitas e, talvez, a maioria das mulheres perdem uma quantidade de sangue no momento do parto suficiente para satisfazer os critérios de diagnóstico para HPP. Alternativamente, HPP tem sido definida como uma queda de 10% ou mais no hematócrito inicial[21].

As estimativas de perda de sangue pós-parto, calculadas pela quantificação visual subjetiva pelos profissionais de saúde, não são exatas na prática clínica, nem sempre refletindo a magnitude do problema[11]. Alternativas têm sido descritas para melhor quantificação da perda sanguínea pós-parto dentre elas, a utilização de sacos plásticos com um fundo cônico e com marcações em mililitros (ml) [22], sacos plásticos sem marcações em ml e a utilização de absorventes, que posteriormente serão pesados[23].

A quantificação errônea da perda de sangue pode levar a subestimação real dessa perda no pós-parto, retardando o reconhecimento da HPP. Isto é especialmente importante em ambientes de poucos recursos, onde leigas e parteiras tradicionais atendem a partos domiciliares, cuja subestimação da perda sanguínea pode retardar a instituição do tratamento, o que piora consideravelmente o prognóstico da parturiente[22].

A assistência ao parto assume um papel extremamente relevante, porquanto a maioria das mortes irá ocorrer no período periparto, sendo essencial a intervenção de profissionais qualificados e hospitais bem aparelhados para o tratamento das emergências obstétricas[8].

Terceiro período do parto: manejo ativo versus manejo passivo

O manejo fisiológico ou passivo, também chamado de conduta expectante, compreende em esperar por sinais de descolamento, permitindo que a placenta se desprenda espontaneamente ou por ação da gravidade, ou ainda, através da estimulação mamilar, que libera ocitocina endógena[24].

O manejo ativo se constitui na utilização de medidas com intuito de diminuir a duração do terceiro período do parto e a perda sanguínea que ocorre durante seu curso. Para o manejo ativo, tem sido proposto um conjunto de intervenções que incluem o uso de uterotônicos, ligadura imediata do cordão umbilical, massagem uterina e tração controlada do cordão[25].

A revisão sistemática da Cochrane sugere que encurtar o terceiro período do parto e garantir que o útero esteja bem contraído durante este tempo pode diminuir a perda de sangue e a incidência de hemorragia[8]. Os autores concluíram que a conduta ativa na terceira fase do parto, traz benefícios para as mulheres com sangramento em termos de redução da grave perda de sangue (RR= 0,34; IC95% 0,14-0,87)[8] e a incidência de transfusões de sangue[8,26].

Uma consideração importante sobre a metodologia dos estudos disponíveis, e inclusive aqueles incluídos na revisão sistemática da Cochrane, é que a forma de se estimar a perda sanguinea difere de estudo para estudo e é geralmente imprecisa. Não se dispõem de estudos em que a perda tenha sido avaliada de forma objetiva, ou seja, a perda volêmica é avaliada de forma estimada, de forma subjetiva.

Outro ponto importante é o fato da maioria dos estudos utilizarem um conjunto de medidas chamado Manejo ativo, que muitas vezes é composto por diferentes componentes, o que torna difícil a estimação da magnitude da importância de cada uma das medidas.

Manejo Ativo

Uso Profilático de Uterotônicos (ocitocina, ergometrina, misoprostol)

As drogas mais utilizadas como uterotônicos são a ergometrina (derivada do ergot) e a ocitocina[27]. A ergometrina é um alcalóide que estimula as contrações uterinas [28]. A ocitocina é um hormônio sintetizado de forma endógena que atua sobre os receptores hormonais no organismo para induzir as contrações do útero que é um órgão composto por tecido muscular liso[29], diminuindo dessa forma sanguínea pós-parto.

A administração da ocitocina sintética profilática objetivando prevenir a HPP é usada durante o terceiro período do parto[6,27]. A Organização Mundial de Saúde (OMS) recomenda utilizá-la como droga de primeira escolha para prevenção da HPP[6]. A recomendação é que seja aplicada por via intramuscular, na dose de 10 UI[6,27], havendo ainda controvérsias sobre o momento exato de sua administração. Enquanto alguns estudos sugerem benefícios com a administração logo após a saída do neonato, outros demonstram maior redução do risco de hemorragia pós-parto após o desprendimento dos ombros ou espáduas. Essa questão precisa ser elucidada em estudos posteriores[11] .

Uma revisão sistemática publicada na biblioteca Cochrane[11] com o objetivo de comparar os efeitos da ocitocina e da associação da ergometrina e ocitocina, mostrou que a combinação ocitocina-ergometrina é mais efetiva para a redução da perda sanguínea maior que 500 mL em relação à ocitocina isolada, sem efeitos sobre perdas acima de 1.000 mL, associando-se com aumento do risco de náuseas, vômitos e elevação da pressão arterial com a utilização da ocitocina-ergometrina[11]. Esses riscos precisam ser considerados antes de se decidir pelo uso da combinação ocitocina-ergometrina ou ocitocina isolada para manejo ativo do terceiro estágio do parto.

A ergometrina isolada também reduz a hemorragia maior que 500 mL (RR=0,38; IC95% 0,21-0,69), porém aumenta o risco de náuseas e vômitos (RR=11,81;IC95% 1,78-78,28), hipertensão arterial (RR= 2,60; IC95% 1,03-6,57), além de dor (RR=2,53; IC95% 1,34-4,78) é o que mostra uma Revisão Sistemática da biblioteca Cochrane que objetivou determinar a eficácia e a segurança da conduta expectante com o uso da ergometrina isolada na terceira fase do parto[30].

Outra revisão sistemática (incluindo 14 ensaios clínicos randomizados e envolvendo 8600 mulheres) também publicada na biblioteca Cochrane compara a utilização da ocitocina a nenhum uterotônico, da ocitocina-ergometrina a ocitocina e a ergometrina isolada. Tal estudo evidenciou que o uso isolado de ocitocina profilática é efetivo em termos de redução da hemorragia pós-parto maior que 500 mL (RR=0,50; IC95%: 0,43-0,59) e maior que 1.000 mL (RR=0,61; IC95%: 0,44-0,87) e da necessidade de ocitocina terapêutica (RR= 0,50; IC95% :0,39-0,64)[31].

Em relação ao manejo ativo do terceiro estágio com uterotônicos, há evidências de que ocorre redução da perda sanguínea no parto e de hemorragia pós-parto, além de redução do terceiro estágio (em torno de 10 minutos)[1]. Em contrapartida, existe aumento do risco de náuseas maternas, vômitos e elevação da pressão arterial ligados ao uso da ergotamina. Portanto, os autores sugerem que esses efeitos adversos devam ser considerados e que essas evidências não devem ser extrapoladas para partos não-hospitalares[1] .

Além da ergometrina e da ocitocina, o misoprostol tem sido sugerido como uma alternativa na prevenção da HPP, uma vez que é um análogo da prostaglandina E1, atuando como um agente uterotônico efetivo[23]. Relaxa o músculo liso da cérvice e facilita a dilatação, ao mesmo tempo em que permite o acréscimo do cálcio intracelular, promovendo uma contração uterina eficaz e suave. Todos estes mecanismos permitem o

progressivo esvaecimento e a dilatação cervical, concomitante ao discreto aumento inicial da atividade uterina[32] .

Dois ensaios clínicos mostram que o uso de misoprostol é efetivo em termos de redução do risco da hemorragia pós-parto. Esses ensaios foram realizados no continente asiático (incluídas 2017 mulheres) e no continente africano (incluídas 661 mulheres), respectivamente, e utilizaram o misoprostol no terceiro período, na posologia de 400 µg via oral[33] e o outro na posologia de 600 µg via sublingual[29]. Todavia, um outro Ensaio Clínico Randomizado (ECR) multicêntrico (incluídas 1103 mulheres) utilizou 400 µg de misoprostol por via oral e não encontrou benefício nesse manejo[34].

Uma revisão sistemática que estudou a eficácia do uso profilático do misoprostol por via oral ou sublingual, na dose de 600 µg, comparativamente ao placebo ou ocitocina mostrou que é uma alternativa eficaz na redução da HPP grave e transfusão de sangue (RR=0,31; IC95%: 0,10-0,94)[35].

Na prática, a ocitocina é a droga de escolha por ser mais barata e mais efetiva que misoprostol para redução da hemorragia pós-parto[35], sendo a droga de escolha para prevenção de HPP[6], a ocitocina isolada é suficiente para o manejo ativo do terceiro estágio do parto[36].

Clampeamento Precoce do Cordão Umbilical

O clampeamento precoce do cordão umbilical consiste em clampear o cordão umbilical imediamente após a saída do neonato[37]. Essa conduta, que fazia parte do conjunto de intervenções do manejo ativo do terceiro período do parto, não é mais recomendada, uma vez que evidências recentes comprovam os benefícios da ligadura

tardia do cordão para o recém-nascido que apresenta boa vitalidade[38]. Adiar esse pinçamento entre 30 e 120 segundos está associado a uma redução no risco de hemorragia intraventricular e menor necessidade de transfusão de sangue[37]. Além de promover ótima transição neonatal, prover oxigênio, nutrientes e volume de sangue adicional ao neonato. Autores mostram, que o clampeamento tardio do cordão aumenta o volume sanguíneo do recém-nascido em 30%, entretanto, o clampeamento precoce previne icterícia (decorrente do aumento da viscosidade do sangue), a taquipnéia transitória e a policitemia (hematócrito > 60-70%) e por sua vez, aumenta a frequência de anemia durante a infância[38,39]. Mesmo assim, a recomendação atual é o clampeamento tardio do cordão, levando em consideração que esses desfechos não são frequentes e quando existentes são superados pelos recém-nascidos de baixo risco[27]. Em se tratando de recém-nascidos prematuros, ou seja, gestação menor que 37 semanas completas, uma revisão sistemática da biblioteca Cochrane mostra que atrasando pinçamento do cordão umbilical entre 30 e 120 segundos parece ser melhor do que dentro de 30 segundos, já que está associado a uma redução no risco de hemorragia intraventricular (RR=1,74; IC95% 1,08-5,67) e menor necessidade de transfusão de sangue[37]. Os primeiros estudos incluídos nas revisões sistemáticas da Cochrane incluíam essa medida.

Tração controlada do cordão e Massagem em Fundo Uterino

A tração controlada do cordão e a massagem em fundo uterino constituem manobras que podem ser feitas de forma conjunta ou separadamente e que envolvem respectivamente, a tração do cordão umbilical, mantendo para cima o mesmo e colocando a mão oposta sobre o abdome logo acima da sínfise púbica[27,40] e a massagem em fundo do útero, ou seja, a realização da fricção do abdome. Essas ações atuam liberando

prostaglandina local, reduzindo a perda sanguínea, e consequentemente, a HPP, e a necessidade de terapêutica adicional com uterotônicos[41].

Uma revisão sistemática que alocou aleatoriamente 200 mulheres comparando a realização da massagem uterina com a conduta expectante, depois da utilização de ocitocina na terceira fase do parto mostrou que a massagem uterina (a cada 10 minutos durante 60 minutos após o nascimento) juntamente com ocitocina é eficaz na redução da perda sanguínea média e da necessidade de uterotônicos adicionais (RR=0,20; IC95% 0,08-,0,50). Apesar de ser eficaz e trazer vantagens importantes, por ser barato e não requerer acesso à medicação ou outros serviços especializados, além de poder ser usado em qualquer local em que as mulheres dão à luz; apresenta como desvantagens o uso do tempo do pessoal e o desconforto causado nas mulheres[41].

Não há relatos de complicações graves associadas com esta técnica, embora os autores recomendem novos estudos com um número de mulheres maior para estimar os efeitos da massagem com e na ausência de ocitocina[41].

Em se tratando da utilização da tração controlada do cordão, um ensaio clínico randomizado mostrou que essa conduta pode reduzir a perda de sangue pós-parto em comparação com a conduta expectante. Embora o efeito observado seja benéfico sobre a perda de sangue e HPP os achados sustentam a realização de um grande ensaio para determinar se a tração controlada do cordão pode, de fato, prevenir hemorragia pós-parto[42]. Por outro lado, deve-se atentar para o risco de inversão uterina facilitada por essa manobra. Apesar de rara, é uma ocorrência grave, relacionada à inexperiência e negligência do profissional em praticar tal estratégia[27].

Em contrapartida, uma revisão sistemática da biblioteca Cochrane com o objetivo de determinar a eficácia da pressão uterina comparando a tração controlada do cordão,

não identificou estudos randomizados controlados, como parte de conduta ativa da terceira fase do parto[40].

Drenagem placentária

Uma forma alternativa de acelerar a dequitação é a drenagem placentária a qual consiste na retirada da pinça do cordão umbilical da extremidade materna, que durante o parto foi fixado para separação do cordão entre o recém-nascido e a mulher, onde a partir daí o sangue da placenta será drenado espontaneamente até o delivramento[25]. A drenagem placentária tem sido sugerida como uma forma de minimizar o impacto do terceiro período do parto para as mulheres. Acredita-se que drenar o sangue da placenta irá reduzir seu volume, permitindo que o útero se contraia e retraia, auxiliando dessa forma no delivramento[25].

Alguns autores afirmam que a drenagem placentária é um método seguro e que não aumenta o risco de complicação pós-parto[24,25]. É uma conduta mais fisiológica e menos intervencionista, e evita procedimentos desconfortáveis logo após o nascimento quando a mãe pretende concentrar-se no seu bebê[25].

Pode ser uma alternativa eficaz para redução na duração do terceiro período do parto, concluem os autores[24]. É o que mostra um ensaio clínico randomizado que teve como objetivo avaliar o efeito da drenagem placentária sobre a duração da terceira fase do parto, e mostrou que em 99 casos (49 casos ao grupo de estudo e 50 casos ao grupo controle). Observou-se uma redução significativa na duração do terceiro estágio após a drenagem (5,1 ± 2,4 minutos versus 7,0 ±6,1 minutos). Não houve casos de HPP, atonia uterina, choque hipovolêmico ou a necessidade de transfusão sanguínea em nenhum dos grupos. Nesse estudo, não foi avaliada a perda volêmica[24].

A drenagem placentária associada à tração controlada do cordão também foi avaliada em um ECR realizado na França, que comparou 239 mulheres que tiveram drenagem placentária acrescido de tração controlada do cordão, com 238 mulheres com a conduta expectante. Tal estudo evidenciou nenhuma diferença significativa nos dois grupos com relação à incidência da remoção manual da placenta retida ou hemorragia pós-parto, porém os valores medianos da duração da terceira fase do parto e a queda nos níveis de hemoglobina foram significativamente menores no grupo da drenagem placentária[43].

Outro ensaio clínico reforça que a drenagem placentária é efetiva na redução do terceiro período parto. Nesse ensaio realizado na Índia, foram envolvidas 958 mulheres que tiveram parto transpelviano e randomizadas para drenagem placentária (478 mulheres) ou para tração controlada do cordão umbilical (480 mulheres). Os autores observaram que a duração média do terceiro período foi de 3,24 min e 3,20 min no grupo da drenagem placentária, em contraste com 8,57 min e 6,20 min no método de tração controlada em primíparas e multíparas, respectivamente[44]. Vale salientar, que nesse estudo a perda volêmica não foi avaliada tanto no grupo da drenagem placentária quanto no grupo da tração controlada do cordão umbilical.

Uma revisão sistemática publicada na biblioteca Cochrane que objetivou avaliar os efeitos da drenagem placentária com e sem o uso profilático de ocitocina no terceiro período, incluiu três estudos envolvendo 1.257 mulheres e observou que a drenagem placentária reduz o terceiro período do parto cerca de três minutos (IC95%: -4,04-1,66). Não houve diferença na remoção manual da placenta ou o risco de hemorragia pós-parto ou incidência de transfusão de sangue. Os ensaios incluídos não relataram dor materna ou desconforto durante a terceira fase do parto[25].

Apesar de ser demonstrado que a drenagem placentária, tanto individualmente, quanto associada sinergicamente a outras práticas, diminui o tempo do terceiro período do parto, ainda não é utilizada de rotina na prática profissional. Existem muitas incógnitas quanto ao manejo ideal durante o terceiro período. Os estudos mostram que a drenagem é eficaz na diminuição do terceiro período, porém a perda volêmica não é abordada de forma objetiva. Geralmente, a quantificação volêmica é feita de forma estimada pelos profissionais, sem exatidão.

Vários estudos sobre o efeito do manejo ativo do terceiro período do parto na redução do sangramento pós-parto têm sido realizados em diferentes países. Em relação a sua efetividade, quando comparado ao manejo expectante, observa-se que a conduta ativa é mais eficaz, porém esse manejo pode indicar combinações de diferentes intervenções com diferentes resultados[1,24,31]. Daí a dificuldade de se avaliar os vários resultados e definir a melhor estratégia entre manejo ativo e conduta conservadora na dequitação placentária[27] porém, atualmente, a recomendação mais segura e eficiente é de usar ocitocina para prevenção de hemorragia pós-parto[36].

Diante das evidências, devemos investigar medidas complementares às drogas para a redução de hemorragia durante o terceiro período do parto e uma das medidas a ser avaliada é a drenagem placentária. É uma manobra inócua, de fácil realização, sem custo, que não utiliza mais ainda medicamentos durante o parto, e dessa forma, se for demonstrado que ela aumenta a efetividade do manejo, seria de extrema relevância para assistência à mulher, podendo ser acrescentada aos cuidados preestabelecidos como rotina.

II. JUSTIFICATIVA

No terceiro período do parto, as evidências sugerem que a realização da drenagem placentária resulta em redução em seu tempo de desprendimento, diminuindo o risco de sangramento excessivo. Contudo, a perda volêmica ocorrida neste período ainda não foi avaliada de forma objetiva, ou seja, é avaliada de forma estimada, de forma subjetiva. A drenagem placentária poderia ser usada de forma complementar às manobras do manejo ativo devido aos benefícios maternos, porém esse manejo não é adotado como protocolo institucional, dessa forma surgiu a necessidade de realizar essa pesquisa.

A pesquisa será realizada no setor do Instituto de Medicina Integral Prof. Fernando Figueira – IMIP e no Hospital Petronila Campos, ambos em Pernambuco, onde as mulheres de baixo risco são assistidas durante o trabalho de parto/parto e puerpério, locado no terceiro andar da maternidade, a assistência é prestada por enfermeiros obstetras e por residentes de enfermagem, os quais possuem a expertise para colaborar na realização da coleta, levando em consideração que não levará a nenhum ônus para as instituições.

O tema abordado é novo no sentido de verificar a perda sanguínea, pois essa não foi avaliada de forma eficaz em nenhum outro estudo e assim poderá contribuir para a literatura já disponível sobre o tema, podendo ser incluído na revisão sistemática disponível para elucidar esse aspecto que ainda persiste por ser esclarecido.

A pesquisa só será iniciada após aprovação do Comitê de Ética em Pesquisa e seguirá as normas da Resolução 196/96. A paciente, caso concorde em participar, deverá assinar o Termo de Consentimento Livre e Esclarecido (TCLE), após a sua leitura e os possíveis esclarecimentos. Será garantido à cada participante o direito de desistir do estudo a qualquer momento, não havendo prejuízo ou interferência na sua terapêutica. O procedimento não envolve riscos, nem individuais nem coletivos (beneficência), haverá garantia de que danos previsíveis serão evitados (não maleficência).

O estudo é relevante, uma vez que as conclusões poderão ser de grande utilidade para a conduta clínica, em se tratando de um método não invasivo e sem custo que pode contribuir para a redução do grande problema representado pela hemorragia pós-parto. Além de reduzir os custos hospitalares com medicações adicionais que poderiam ser utilizadas na vigência de hemorragia pós-parto, também permitirá reduzir o excesso de intervenções no manejo do terceiro estágio do parto. O resultado da pesquisa pode vir a alterar condutas e protocolos institucionais e diretrizes nacionais e internacionais, além de servir de subsídio para novas pesquisas.

III. HIPÓTESES

3.1.Desfechos Primários:

- A duração do terceiro período do parto e o volume da perda sanguínea na primeira hora pós-parto é menor no grupo experimental.

3.2.Desfechos secundários:

- O risco de Hemorragia pós-parto (>500ml) e Hemorragia pós-parto grave (≥1000ml) na primeira hora é menor nas mulheres submetidas à drenagem placentária;
- Devido a menor perda volêmica nas mulheres submetidas à drenagem placentária há um risco diminuído de Hemoglobina materna com 24-48h pós-parto menor que 8g/dl, de necessidade de hemotransfusão;
- O hematócrito (pré e pós-parto) é maior nas mulheres submetidas à drenagem placentária devido a menor perda volêmica no terceiro período do parto;
- No grupo experimental há redução na necessidade de uso terapêutico de ocitócitos na primeira hora e dentro de até 24 h pós-parto;
- Nas mulheres submetidas à drenagem placentária o terceiro período do parto é menor que 30 minutos;
- Terceiro período maior que 60 minutos é indiferente em ambos os grupos;
- A necessidade de curagem uterina, remoção manual da placenta de curetagem uterina é similar em ambos os grupos;
- Sintomas de anemia até 48 h pós-parto(tontura, cefaleia, cansaço) são menos evidentes com a utilização da drenagem placentária;
- Satisfação materna com o manejo do terceiro período é indiferente em ambos os grupos.

IV. OBJETIVOS

4.1. OBJETIVO GERAL

Determinar a efetividade da drenagem placentária comparada à manutenção da extremidade materna do cordão clampeada no terceiro estágio do parto.

4.2. OBJETIVOS ESPECÍFICOS

Em mulheres no terceiro estágio submetidas à drenagem placentária vs. clampeamento da extremidade materna do cordão umbilical, comparar:

4.2.1.Desfechos Primários:

- Duração do terceiro período do parto
- Volume da perda sanguínea na primeira hora pós-parto

4.2.2.Desfechos secundários:

- Hemorragia pós-parto (>500ml) na primeira hora
- Hemorragia pós-parto grave (≥1000ml) na primeira hora
- Hemoglobina materna com 24-48h pós-parto menor que 8g/dl
- Diferença de hematócrito (pré e 24-48h pós-parto)
- Necessidade de hemotransfusão
- Necessidade de uso terapêutico de ocitócitos na primeira hora pós-parto
- Necessidade de uso terapêutico de ocitócitos dentro de até 24 h pós-parto
- Terceiro período maior que 30 minutos
- Terceiro período maior que 60 minutos
- Necessidade de curagem uterina
- Remoção manual da placenta
- Necessidade de curetagem uterina
- Sintomas de anemia até 48 h pós-parto (tontura, cefaleia, cansaço)

- Satisfação materna com o manejo do terceiro período

V. MÉTODOS

5.1. Desenho do estudo

Será realizado um ensaio clínico randomizado aberto, comparando mulheres no terceiro estágio do parto que serão submetidas ou não à drenagem placentária.

5.2. Local do estudo

O estudo será realizado no IMIP e no Hospital Petronila Campos onde as mulheres de baixo risco são assistidas durante o trabalho de parto/parto e puerpério, locais onde são atendidas gestantes de baixo risco obstétrico, durante o pré-parto, parto e pós-parto (PPP).

5.3. Período de coleta de dados

A coleta dos dados será realizada no período compreendido entre os meses de agosto de 2012 a maio de 2013.

5.4. População do estudo

Inclui todas as mulheres de baixo risco obstétrico, a termo, com feto vivo, em trabalho de parto atendidas nos setores de baixo risco durante o pré-parto/parto e puerpério.

5.5. Amostra

5.5.1. Amostragem

Será obtida uma amostra não probabilística, de conveniência, composta pelas gestantes atendidas, no período do estudo que preencherem os critérios de elegibilidade. As pacientes elegíveis para o estudo serão randomizadas em dois grupos: com e sem drenagem placentária.

5.5.2. Tamanho da amostra

O cálculo do tamanho da amostra foi feito através da seguinte fórmula para variáveis dicotômicas:

$$\mathbf{ZN = 4\frac{(Z\alpha + Z\beta)^2\,\bar{p}(1-\bar{p})}{(p_C - p_I)^2}}$$

Onde:

ZN = número total da amostra (N = número de participantes por grupo)

$Z\alpha$ = escore Z do erro alfa (1,96 para um erro alfa de 5%)

$Z\beta$ = escore Z do erro beta (1,282 para um poder de 90%)

p = $(p_C + p_I)/2$

p_C = proporção no grupo do controle

p_I = proporção no grupo de intervenção[45.]

Para este cálculo foi utilizado o programa OpenEpi versão 2.3 (Atlanta, GA), de domínio público. O parâmetro utilizado para cálculo do tamanho da amostra foi a duração do terceiro período do parto. Um ensaio clínico randomizado publicado em 2009, mostrou que há redução no terceiro período do parto com a realização da drenagem placentária (5,1± 2,4 minutos versus 7,0 ±6,1 minutos)[24].

Considerando um nível de significância de 5% e um poder de 80%, seriam necessárias 188 mulheres para evidenciar diferença entre os grupos. Prevendo-se eventuais perdas, este número foi aumentado para 226 puérperas, sendo 113 em cada grupo.

5.6. Critérios e procedimentos para seleção das participantes

5.6.1. Critérios de Inclusão

- Parturientes de baixo risco obstétrico;
- Gestação única a termo (37 a 42 semanas);
- Parto assistido nos serviços onde o estudo será conduzido, onde as mulheres de baixo risco são assistidas durante o trabalho de parto/parto e puerpério;
- Feto vivo.

5.6.2. Critérios de exclusão

- Mulheres incapazes;
- Mulheres que concordaram em participar, assinaram o TCLE, porém não evoluíram para cesariana e fórceps.

5.6.3. Procedimentos para Captação e Acompanhamento das Participantes

Antes de iniciar a coleta de dados, será realizada uma sensibilização de todos os profissionais, promovendo a divulgação do projeto pelos pesquisadores responsáveis, por meio de cartazes e comunicação oral, explicando os objetivos, a justificativa e os métodos da pesquisa. Esses profissionais previamente serão treinados pelos pesquisadores.

Esses profissionais identificarão as possíveis mulheres candidatas ao estudo. Uma vez identificada uma possível participante, o pesquisador/assistente irá aplicar uma lista de checagem (Apêndice 1) para confirmar o cumprimento dos critérios de elegibilidade. Caso a gestante preencha os critérios, será abordada pelo pesquisador/assistente, e receberá informações acerca dos motivos da pesquisa, e sua importância, sendo

convidada a ingressar nesta. As gestantes serão ainda informadas que, caso não aceitem participar do estudo, o seu acompanhamento será realizado segundo a rotina habitual do serviço por uma equipe capacitada, sem nenhum prejuízo de qualquer ordem.

Caso concordem voluntariamente em participar, será solicitada a assinatura do Termo de Consentimento Livre e Esclarecido (TCLE) (Apêndice 2), após leitura cuidadosa e explicações pelo pesquisador/assistente. Posteriormente, será coletada uma amostra de sangue pelo profissional do laboratório antes do parto e encaminhada para análise laboratorial. Todas as participantes terão assistência ao trabalho de parto realizada conforme a rotina do serviço e imediatamente após o parto serão abertos os envelopes lacrados contendo o grupo para o qual cada participante será alocada (Figura 1). A coleta será realizada pelos pesquisadores, enfermeiros obstetras do setor durante o dia, noite e fins de semana. Esses profissionais serão treinados antes do início da pesquisa. Após 24-48 h será coletada uma nova amostra de sangue pelo funcionário do laboratório e encaminhada para análise laboratorial.

5.7. Fluxograma de captação e acompanhamento das participantes

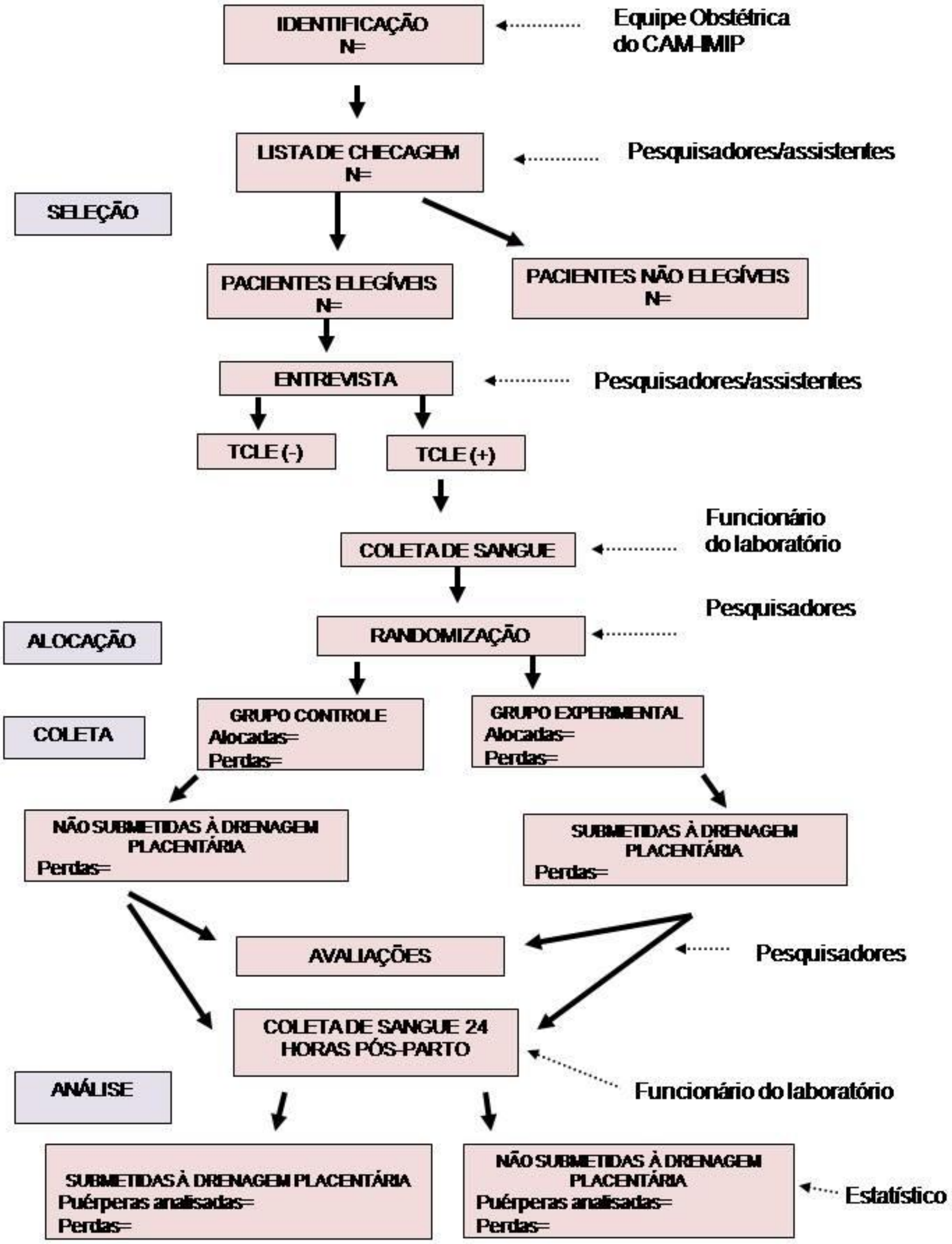

Figura 1. Fluxograma de captação e acompanhamento dos participantes (Fluxograma CONSORT)[46].

5.7.1. Procedimentos para randomização

A randomização para realização ou não da drenagem placentária será feita de acordo com uma lista de números randômicos previamente gerada em computador, utilizando-se o programa Random Allocation Software versão 1.0.

A partir dessa lista, serão preparados envelopes numerados de um a 226, sendo que cada número, de acordo com a lista de randomização, corresponderá à alocação da paciente no grupo experimental ou controle (GE ou GC). Estes envelopes serão preparados por um colaborador do projeto, que não fará parte da coleta dos dados.

A paciente será convidada a participar do estudo e caso concorde, assinará o Termo de Consentimento Livre e Esclarecido (Apêndice 2). Após o parto, aquelas que tenham concordado e assinado o TCLE e que tenham evoluído com parto vaginal espontâneo receberão o envelope lacrado e numerado contendo dentro a informação sobre sua alocação, sendo esse anexado ao prontuário. Somente então será realizada a abertura do envelope lacrado que contém o grupo selecionado para aquela participante, garantindo-se a ocultação da alocação. Os grupos serão classificados da seguinte forma:

GE (grupo experimental): submetido à drenagem placentária

GC (grupo controle): não submetido à drenagem placentária

As mulheres que assinaram o TCLE e que evoluíram para cesariana e parto a fórceps serão excluídas do estudo não recebendo dessa forma o envelope lacrado e numerado.

5.8. Variáveis de análise:

5.8.1. Variável Independente:

- Drenagem placentária: sim ou não

5.8.2. Variáveis Dependentes:

5.8.2.1. Desfechos primários:

- Duração do terceiro período do parto;
- Volume da perda sanguínea uma hora pós-parto

5.8.2.2. Desfechos secundários:

- Hemorragia pós-parto (>500ml) na primeira hora
- Hemorragia pós-parto grave (>1000ml) na primeira hora;
- Hemoglobina materna com 24-48h pós-parto menor que 8g/dl;
- Diferença de hematócrito (pré e 24-48 h pós-parto);
- Necessidade de hemotransfusão;
- Dor abdominal no puerpério;
- Necessidade de uso terapêutico de ocitócitos na primeira hora pós-parto;
- Necessidade de uso terapêutico de ocitócitos dentro de até 24 h pós-parto
- Terceiro período maior que 30 minutos;
- Terceiro período maior que 60 minutos;
- Necessidade de curagem uterina;
- Remoção manual da placenta;
- Necessidade de curetagem uterina;
- Sintomas de anemia até 48 h pós-parto (tontura, cefaleia, cansaço);
- Satisfação materna com o manejo do terceiro período.

5.8.2.3. Variáveis descritivas:

- Idade materna (anos);
- Estatura materna (metros);

- Peso (quilos);
- IMC;
- Raça;
- Procedência;
- Escolaridade;
- Situação marital;
- Renda familiar per capita;
- Número de gestações;
- Paridade;
- Tipos de partos anteriores;
- Postura durante o parto;
- Episiotomia;
- Laceração espontânea;

5.9. Definição e operacionalização de variáveis

5.9.1. Termos

- Terceiro período do parto – é o período compreendido entre o nascimento do bebê até a expulsão da placenta[1].
- Drenagem placentária – consiste no despinçamento do cordão umbilical, que durante o parto foi fixado para separação do mesmo com a placenta, para permitir a drenagem sanguínea[25].

5.9.2. Critérios de Inclusão

- Parturientes de baixo risco – início do trabalho de parto espontâneo no termo, sem nenhum fator de risco identificado, permanecendo nesse quadro durante todo o processo do parto[47].
- Gestação a termo – gestações entre a 37ª e 42ª semana de gravidez de único feto, calculada a partir da data da última menstruação (DUM), desde que conhecida e confiável, confirmada pelo primeiro exame ecográfico. Quando a paciente não souber referir a DUM e/ou houver discordância com o exame ultrassonográfico, a datação da gestação será realizada com base na primeira ultrassonografia.
- Mulheres de baixo risco assistidas durante o trabalho de parto/parto e puerpério.
- Feto vivo – ausculta fetal positiva na admissão determinada pelo estetoscópio de Pinard ou sonar Doppler, e confirmada pela ultra-sonografia quando a ausculta, inicialmente, for negativa.

5.9.3. Critérios de Exclusão

- Mulheres incapazes: menores de 18 anos, deficiente mental e indígenas;
- Mulheres que concordaram em participar, assinaram o TCLE, porém evoluíram com parto cesariana ou parto instrumental a fórceps;
- Parto instrumental a fórceps – parto auxiliado pelo uso de um instrumento metálico, semelhante a uma pinça, constituído por dois braços articulados em forma de colher, que irão segurar a cabeça do bebê e tracioná-la para fora do canal vaginal;
- Cesárea – é o ato cirúrgico que consiste em incisar o abdome e a parede do útero gestante para libertar o concepto aí desenvolvido[48].

5.9.4. Variáveis do Estudo:

5.9.4.1. Variáveis descritivas

- Idade materna (anos) – variável numérica contínua (podendo ser posteriormente categorizada), expressa em anos completos, conforme informação da paciente, no momento de sua seleção para ingressar no estudo.
- Estatura materna (metros) – variável numérica contínua (podendo ser posteriormente categorizada), expressa em metros, indicando sua altura. Informação será colhida pelo pesquisador/assistente ou no prontuário, no momento de sua seleção para ingressar no estudo.
- Peso (quilos) – variável numérica contínua (podendo ser posteriormente categorizada), expressa em quilos, indicando o peso da paciente no momento que ingressou no estudo. Informação será colhida pelo pesquisador/assistente ou no prontuário da paciente.
- Índice de Massa Corpórea (IMC) – calcula-se o IMC a partir da altura em metros e o peso, através da fórmula: $peso/altura^2$ .variável numérica contínua, podendo ser posteriormente categorizada para fins de análise em: *baixo peso*, *peso adequado*, *sobrepeso* e *obesidade*, adotando-se os níveis de corte proposto por Atalah *et al* (ANEXO 1).
- Raça – variável categórica policotômica. Considerando que o estudo é prospectivo e o pesquisador poderá falar com as mulheres, as mesmas irão autodefinir sua raça.
- Procedência – variável categórica policotômica, indicando o lugar onde a mulher reside, podendo ser: Recife e região metropolitana, interior de Pernambuco ou outros estados. Conforme informação da paciente.
- Escolaridade – variável numérica discreta, representando o nível de escolaridade da mulher ou puérpera, expressa em anos completos e aprovados de estudo. Pode ser categorizada posteriormente para análise. Conforme informação da paciente.

- Situação marital – variável categórica dicotômica, expressando a presença ou ausência do companheiro. Conforme informação da paciente.
- Renda familiar per capita – variável numérica contínua (podendo ser posteriormente categorizada para análise) revelando, em reais, a renda por pessoa da família em que a mãe está inserida. Conforme informação da paciente.
- Número de gestações – variável numérica discreta, correspondendo ao número de gestações, incluindo a atual, tendo vindo a termo ou não. Conforme informação da paciente.
- Paridade – variável numérica discreta, correspondendo ao número de partos anteriores concluídos, conforme informação da paciente.
- Parto anterior – variável categórica dicotômica, do tipo sim ou não, descrevendo a presença ou não de parto anterior. Conforme informação da paciente.
- Tipos de parto anteriores – variável numérica, correspondendo ao número de partos transvaginais anteriores e número de cesáreas. Conforme informação da paciente.
- Postura durante o parto – variável categórica dicotômica, determinando qual o posicionamento adotado pela mulher no momento do parto. Serão consideradas posição verticalizada ou não verticalizada. Conforme observação do pesquisador/ assistente.
- Episiotomia - variável nominal dicotômica que expressa a realização ou não da episiotomia. Coletada no prontuário ou conforme observação do pesquisador/ assistente. É uma incisão efetuada na região do períneo (área muscular entre a vagina e o ânus) para ampliar o canal de parto e prevenir que ocorra laceração irregular durante a passagem do bebê[48].
- Laceração espontânea – variável categórica dicotômica, do tipo sim ou não, descrevendo se houve algum tipo de laceração perineal ocorrida espontaneamente

no momento do parto. Coletada no prontuário ou conforme observação do pesquisador/ assistente. São lesões traumáticas da vulva, da vagina e do períneo na ausência de episiotomia[49].

5.9.4.2. Variáveis dependentes

- Duração do terceiro período do parto – variável numérica contínua (podendo ser posteriormente categorizada), expressa em minutos, indicando o tempo da saída da placenta (iniciado após a saída do neonato), conforme registrado pelo pesquisador/ assistente.
- Volume da perda sanguínea uma hora pós-parto – variável numérica contínua (podendo ser posteriormente categorizada), expressa em mililitro (ml), indicando a quantidade de volume de sangue perdido na primeira hora pós-parto. Será avaliada pela coleta do sangue drenado no terceiro período em um saco plástico projetado para esta finalidade que posteriormente será pesado e pela pesagem das compressas utilizadas nesse período.

  - Pesagem dos sacos plásticos: quantificada pelo operador de acordo com a pesagem dos sacos plásticos com sangue drenado no terceiro período do parto. O peso em gramas será convertido para mililitros, dividindo o valor em gramas por 1,05(densidade do sangue em gramas por centímetros cúbicos)[25]. Variável numérica contínua expressa em mililitro (ml).
  - Caso seja utilizada compressa e/ou gaze o volume obtido será acrescentado ao volume obtido com a pesagem dos sacos plásticos. Cada pacote de compressa com 05 unidades pesa 12 gramas e cada

pacote de gaze com 05 unidades pesa 08 gramas. As compressas e as gazes utilizadas serão inseridas na seguinte fórmula:

Nº pacote gaze usada × 08 + Nº pacote compressa× 12- peso total de gazes e compressas no fim do parto

O valor obtido em gramas será convertido para mililitros, dividindo o valor em gramas por 1,05(densidade do sangue em gramas por centímetros cúbicos)[25].

- Perda sanguínea avaliada através da diferença de hematócrito – variável numérica contínua expressa em percentual (%), referente à diferença pontual entre o hematócrito antes e após o parto (medido 24 horas de pós-parto) para cada paciente. O hematócrito antes e após o parto será medido de acordo com o método de Wintrobe[50] que consiste na dosagem da parte sólida do sangue (massa total de células) em relação à parte líquida (plasma), medida em pontos percentuais. Em seguida será determinada entre duas medidas e será utilizado o cálculo para estimativa de perda sanguínea:

$$\mathbf{PSA} = \frac{\mathbf{Volemia \times (Hi - Ht)}}{\mathbf{Média\ dos\ hematócritos\ (inicial\ e\ final)}}$$

onde:

- PSA= perda sanguínea avaliada (ml)
- Volemia= constante da equação, considerada como 6000 ml
- Hi=hematócrito antes do parto
- Ht=hematócrito 24-48 h após o parto

- Hemorragia pós-parto (>500 ml) na primeira hora – variável categórica dicotômica, do tipo sim/não. Expressa a presença ou não de hemorragia pós-parto maior que 500 ml. Perda sanguínea mensurada através da pesagem dos sacos plásticos, pesagem das compressas/gazes e diferença de hematócrito pelo pesquisador/ assistente.
- Hemorragia pós-parto grave (>1000ml) na primeira hora – variável categórica dicotômica, do tipo sim/não. Expressa a presença ou não de hemorragia pós-parto maior que 1000 ml. Perda sanguínea mensurada através da pesagem dos sacos plásticos, pesagem das compressas/gazes e diferença de hematócrito pelo pesquisador/ assistente.
- Hemoglobina materna pós-parto menor que 8g/dl – variável categórica dicotômica, do tipo sim/não, expressa a presença ou não da hemoglobina materna 24-48 horas após o parto menor que 8g/dl. Coletada 24-48 horas após o parto e o resultado posteriormente será lançado no sistema da Instituição e resgatado pela pesquisadora.
- Necessidade de hemotransfusão – variável categórica dicotômica, do tipo sim/não. Definida como a realização ou não da transfusão sanguínea após o parto, conforme observação do pesquisador/ assistente.
- Dor abdominal no puerpério – variável categórica dicotômica, do tipo sim/não, correspondente a presença ou não da dor abdominal, conforme informação da paciente.
- Necessidade de uso terapêutico de ocitócitos na primeira hora pós-parto – variável categórica, dicotômica, tipo sim/não, definido como a necessidade de utilização de ocitocina no terceiro período, excetuando-se as duas ampolas de ocitocina utilizadas

de rotina em todas as pacientes atendidas no serviço nesse período. Coletada no prontuário ou conforme pesquisador/ assistente.

- Necessidade de uso terapêutico de ocitócitos dentro de até 24 h pós-parto – variável categórica, dicotômica, tipo sim/não, definido como a necessidade de utilização de ocitocina complementar no terceiro período dentro de até 24 h pós-parto, excetuando-se as duas ampolas de ocitocina utilizadas de rotina em todas as pacientes atendidas no serviço nesse período. Coletada no prontuário ou conforme pesquisador/ assistente.
- Terceiro período maior que 30 minutos – variável categórica dicotômica, do tipo sim ou não, descrevendo se o tempo da saída da placenta foi superior a 30 minutos, conforme observação do pesquisador/ assistente.
- Terceiro período maior que 60 minutos – variável categórica dicotômica, do tipo sim ou não, descrevendo se o tempo da saída da placenta foi superior a 60 minutos, conforme observação do pesquisador/ assistente.
- Necessidade de curagem uterina – variável categórica dicotômica, do tipo sim/não, expressando a realização ou não da curagem uterina, conforme observação do pesquisador/ assistente. Curagem uterina consiste na revisão da cavidade uterina ou na retirada de restos placentários utilizando as mãos.
- Remoção manual da placenta – variável categórica dicotômica, do tipo sim/não, expressa a realização ou não da remoção manual da placenta, conforme observação do pesquisador/ assistente.
- Necessidade de curetagem uterina – variável categórica dicotômica, do tipo sim/não, expressa a realização ou não da curetagem uterina, conforme observação do pesquisador/ assistente e/ou registros encontrados no prontuário. Curetagem

uterina consiste na retirada de restos placentários da cavidade uterina utilizando instrumental.

- Sintomas de anemia até 48 h pós-parto (tontura, cefaleia, cansaço) – variável categórica dicotômica, expressando a presença ou ausência de sintomas de anemia até 48 h pós-parto. Conforme observação do pesquisador/ assistente.
- Satisfação materna – variável categórica policotômica, definindo se a mulher está satisfeita com o manejo do terceiro período. Pode variar entre, muito satisfeita, satisfeita, pouco satisfeita, insatisfeita, muito insatisfeita, de acordo com a escala de faces (Ver item 5.10.6). Conforme observação ou investigação do pesquisador/ assistente.

5.10. Procedimentos, testes, técnicas e exames

5.10.1. Procedimento para o Grupo Experimental

Consiste no desclampeamento do cordão umbilical, que durante o parto foi fixado para separação do mesmo com a placenta, onde permitirá que o sangue da placenta drene livremente em um recipiente apropriado[24], diferente do utilizado para medir o volume perdido.

5.10.2. Procedimentos para o grupo controle

O cordão permanecerá pinçado depois de seu clampeamento, até o desprendimento da placenta.

5.10.3. Avaliação da duração do terceiro período do parto

Para avaliação da duração do terceiro período do parto será utilizado o relógio que existe em cada um dos apartamentos do setor. Em ambos os grupos essa avaliação será iniciada do nascimento do neonato até a saída da placenta.

5.10.4. Avaliação da perda sanguínea na primeira hora pós-parto

- Pesagem dos sacos plásticos: o volume sanguíneo será calculado através da drenagem do sangue que será coletado em um saco plástico projetado para esta finalidade, onde o pesquisador irá colocá-lo sob as nádegas da puérpera imediatamente após o parto e se estenderá para um recipiente apropriado de inox permitindo a drenagem do sangue até a saída da placenta. Posteriormente, esse material será pesado em balança eletrônica onde será obtido o peso em gramas e a partir daí será obtido o volume através da seguinte fórmula:

$$\mathbf{d} = \frac{\textbf{Massa}}{\textbf{Volume}}$$

onde:

- d= densidade sanguínea 1,05 g/cm^3 (gramas por centímetro cúbicos)[25]
- Massa =expressa em g (gramas)
- Volume= expresso em centímetros cúbicos que será convertido em ml (1ml=1 cm^3)

- Caso seja utilizada compressa e/ou gaze o volume obtido será acrescentado ao volume obtido com a pesagem dos sacos plásticos. Cada pacote de compressa com cinco unidades pesa 12 gramas e cada pacote de gaze com cinco unidades pesa 08 gramas. As compressas e as gazes utilizadas serão inseridas na seguinte fórmula:

Nº pacote gaze usada × 08 + Nº pacote compressa× 12- peso total de gazes e compressas no fim do parto

O valor obtido em gramas será convertido para mililitros, dividindo o valor em gramas por 1,05(densidade do sangue em gramas por centímetros cúbicos)[25]

- Diferença de hematócrito: antes do parto e 24-48 horas após o parto será realizada a coleta de 3 ml de sangue da puérpera para avaliar o nível hematócrito no sangue. Esse material será encaminhado ao laboratório, onde posteriormente o resultado do exame será lançado no sistema da instituição e resgatado pelos pesquisadores, o qual sai em média com duas horas. O custo do exame será de responsabilidade das pesquisadoras caso não seja aprovado pela Fundação de Apoio a Pesquisa/FAPE. Caso o resultado do exame tenha alteração importante a mulher receberá os cuidados de acordo com o protocolo da instituição. Esse hematócrito pré e pós-parto será medido de acordo com o método de Wintrobe[50] que consiste na dosagem da parte sólida do sangue (massa total de células) em relação à parte líquida (plasma), medida em pontos percentuais. Em seguida será determinada a diferença entre estas duas medidas e utilizar-se-á o cálculo para estimativa de perda sanguínea:

$$\mathbf{PSA} = \frac{\mathbf{Volemia \times (Hi - Ht)}}{\mathbf{Média\ dos\ hematócritos}}$$

- onde: PSA= perda sanguínea avaliada
- Volemia= constante da equação, considerada como 6000 ml
- Hi=hematócrito antes do parto
- Ht=hematócrito 24-48 h após o parto

5.10.5. Avaliação dos níveis de hemoglobina 24-48 horas após o parto

Após 24 horas do parto será realizada a coleta de 3ml de sangue da puérpera para avaliar o nível de hemoglobina sanguínea. Esse material será encaminhado ao laboratório, onde posteriormente o resultado do exame será lançado no sistema da instituição e resgatado pelos pesquisadores, o qual sai em média com duas horas. O custo do exame será de responsabilidade das pesquisadoras caso não seja aprovado pela Fundação de Apoio a Pesquisa/FAPE Caso o resultado do exame tenha alteração importante a mulher receberá os cuidados de acordo com o protocolo da instituição.

5.10.6. Avaliação da satisfação materna

A paciente será interrogada, 24-48 h pós-parto, pelo pesquisador sobre o nível de sua satisfação com o tratamento que lhe foi aplicado. A escala de satisfação será uma associação entre a escala de faces (FIGURA 2) e a escala numérica. Cada nível será claramente explicado para a paciente da seguinte forma:

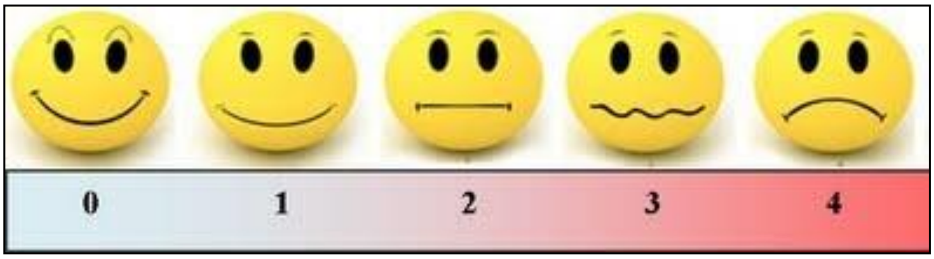

Figura 2: Escala de faces para avaliação da satisfação maternal com o manejo do terceiro período

- 0 – Muito satisfeita.
- 1 – Satisfeita.
- 2 – Pouco satisfeita.
- 3 – Insatisfeita.
- 4 – Muito insatisfeita.

Para fins de análise, a satisfação será recodificada em satisfeita (sim ou não), correspondendo o sim às categorias 0 e 1 acima caracterizadas[51].

5.11. Critérios para descontinuação do estudo

O estudo será interrompido caso sejam identificadas intercorrências secundárias, provenientes do método que cause algum prejuízo físico, moral ou psíquico para as pacientes (extremamente improváveis porque nunca foram descritas).

No caso de um grupo apresentar disparidades evidentes em relação ao outro e essas diferenças tornarem óbvio o prejuízo da drenagem, ou da não drenagem antes mesmo de terminar o ensaio, por questões éticas, este será dado por encerrado e seus resultados divulgados.

Ademais, deve-se ressaltar que também serão respeitados o bem-estar da paciente e a sua vontade em sair do estudo em qualquer fase será soberana e inquestionável.

5.11.1. Comitê de Monitoramento Externo (CME)

O comitê de monitoramento dos dados será um grupo de pesquisadores independentes e externos ao estudo que assegurará a progressão e segurança dos dados e, se necessário, criticará a eficácia e poderá finalizar o ensaio clínico. Apenas o CME poderá dispor de informações quando necessário sobre o cegamento do ensaio clínico.

Membros do CME e suas qualificações

- Maria Inês Bezerra de Melo. Enfermeira Obstetra do IMIP. Doutoranda em Saúde Materno Infantil pelo IMIP.
- Luciana Marques Andreto: Enfermeira Obstetra da Faculdade Pernambucana de Saúde (FPS). Doutora em Nutrição pela UFPE.

- José Natal Figueroa: Estatístico da pós-graduação do IMIP. Membro do Comitê de Ética de Pesquisa do IMIP. Pós-graduando (doutorado) em Saúde Materno Infantil pelo IMIP.
- Carlos Noronha Neto: Ginecologista e Obstetra do IMIP. Mestre em Tocoginecologia pelo Centro de Saúde Amaury de Medeiros (CISAM).
- Aurélio Costa. Ginecologista e Obstetra do IMIP. Doutor em Saúde Materno Infantil pelo IMIP.

Responsabilidades dos Membros do CME

- Luciana Marques Andreto: Coordenadora do grupo. Responsável pela marcação das reuniões semestrais do grupo e coordenar as discussões das reuniões.
- Carlos Noronha Neto: Secretário. Responsável por registrar em ata as discussões das reuniões e suas decisões. Além de solicitar os formulários de coleta e/ou banco de dados ao pesquisador responsável.
- José Natal Figueroa: Estatístico e Aspectos éticos. Responsável pela análise de ínterim quando pertinente e os aspectos éticos envolvidos no estudo.
- Maria Inês Bezerra de Melo: Avaliador. Responsável pelas discussões do grupo e pelo relatório final.
- Aurélio Costa: Avaliador. Responsável pelas discussões do grupo e pelo relatório final.

Conflitos de Interesse dos Membros do CME

- Maria Inês Bezerra de Melos: Não há.
- Luciana Marques Andreto: Não há.
- Carlos Noronha Neto: Não há.

- José Natal Figueroa: Ao final do estudo o mesmo poderá orientar nas análises estatísticas do estudo, se necessário.
- Aurélio Costa: Não há.

Frequência e formato das reuniões do CME

As reuniões serão semestrais em sala reservada na pós-graduação do IMIP, sem o conhecimento dos pesquisadores envolvidos diretamente com o estudo.

Inicialmente será realizada uma análise da frequência dos principais desfechos estudados, comparando-se com a frequência desses desfechos encontrada em revisão sistemática realizadas no IMIP e disponibilizados para os membros do CME. Esta análise será realizada sem se quebrar o sigilo e sem identificar o grupo de tratamento. Em se constatando frequências dos eventos desfavoráveis maiores que o esperado para a amostra estudada, ou se ocorrerem eventos inesperados, o CME deverá sugerir a realização de analise de ínterim.

Fluxo das reuniões do CME

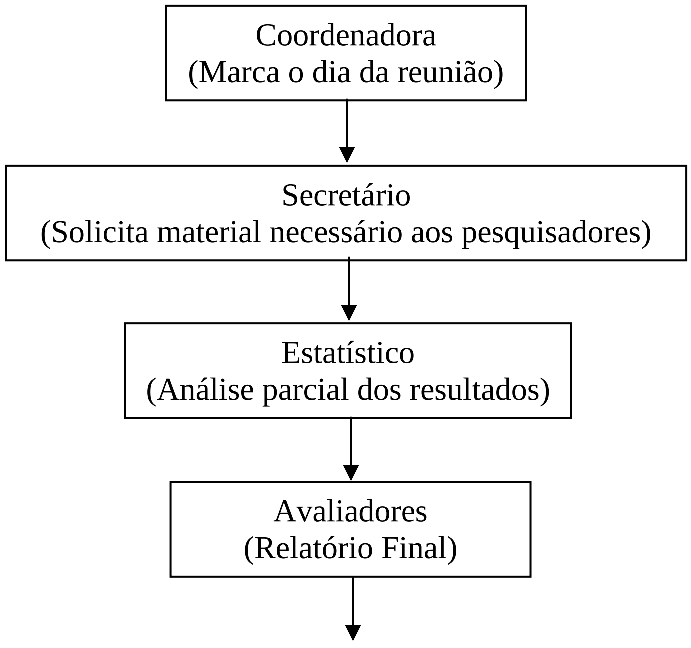

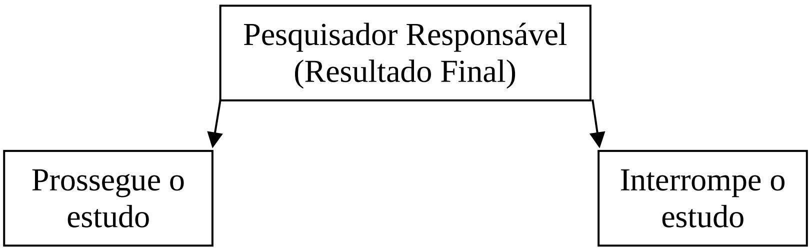

Figura 3. Fluxograma das reuniões do Comitê de Monitoramento Externo.

Plano de análise de ínterim

A análise de ínterim será realizada se alguns dos desfechos apresentarem uma frequência acima do esperado para a amostra estudada, devendo-se quebrar o sigilo para averiguar se um dos grupos está acarretando maiores riscos às participantes e/ou seus conceptos.

5.12. Coleta dos dados

5.12.1. Instrumento para Coleta dos Dados

Para coleta de dados, será utilizado um formulário-padrão, pré-codificado para entrada de dados em computador (Apêndice 3).

5.12.2. Procedimentos para coleta

Após a identificação das gestantes que estiverem de acordo com os critérios de elegibilidade e aceitem participar da pesquisa, tendo assinado o TCLE, informações serão coletadas e preenchidas no formulário. A lista de checagem, assim como o formulário, serão preenchidos pelos pesquisadores.

Após o preenchimento, os formulários serão revisados rigorosamente pelos pesquisadores para a checagem das informações coletadas com as informações constantes

nos prontuários. O tempo transcorrido para coleta de dados, preenchimento adequado de formulários e sua revisão deverá corresponder ao previsto no cronograma.

5.12.3 Controle da qualidade das informações

Os formulários serão checados regularmente e se percebidas inconsistências, estas serão analisadas através de revisão de prontuários ou questionamentos da mulher, se ainda internada.

5.13. Processamento e análise dos dados

5.13.1. Processamento dos Dados

Os dados serão digitados em banco de dados específico, criado no programa estatístico Epi-Info 3.7. A digitação dos dados será realizada após revisão dos formulários, em blocos de dez. Mensalmente este banco de dados será revisado pelo pesquisador principal, obtendo listagem das variáveis e corrigindo eventuais inconsistências ou falta de informações a partir da consulta aos formulários.

Será realizada dupla digitação, em épocas e por pessoas diferentes (o pesquisador principal e um assistente). Após a digitação, os bancos de dados serão comparados. Testes de consistência e tabelas de distribuição de frequências das principais variáveis serão obtidos para correção de eventuais erros. No caso da constatação de inconsistências ou ausência de informações, os formulários serão consultados.

Ao final da digitação, serão novamente obtidas listagens, para a correção final e criação do banco de dados definitivo, que será submetido aos testes de limpeza e consistência das informações e a partir daí será realizada a análise estatística.

5.13.2. Análise dos Dados

A análise dos dados será realizada por um estatístico “cego” colaborador do ensaio, utilizando o programa estatístico Epi-Info 3.7. A análise será por intenção de tratar e será realizada com os grupos identificados como A ou B e somente ao final desta, já preparadas as tabelas, é que esse terá conhecimento sobre qual grupo corresponde a cada letra.

Para avaliação da associação entre a variável independente ou preditora (uso ou não da drenagem placentária) e as variáveis dependentes (desfechos) serão construídas tabelas de dupla entrada. Para as variáveis numéricas contínuas de distribuição normal, será usado o teste “t” de Student, caso não ser verifique a normalidade da distribuição (teste de Kolmogorov-Smirnov), será utilizado o teste de Mann-Whiney. Para as variáveis categóricas, serão utilizados os testes qui-quadro de associação (Pearson) e teste exato de Fisher, quando pertinente (um dos valores esperados menor que cinco). Todos os valores de p adotados serão bicaudados. Será calculada a Razão de Risco (RR) como medida do risco relativo, bem como o Intervalo de Confiança a 95% (IC 95%). À categoria de referência será atribuído o valor padrão de 1,0. Será calculado o Número Necessário para Tratar e obter um benefício (NNT) e o Número Necessário para Tratar e obter um malefício (NNH), com os seus respectivos intervalos de confiança a 95%, para os desfechos que demonstrarem associação estatisticamente significante com a drenagem placentária. Esse cálculo será realizado utilizando-se o programa de domínio público EBM Calculator .

5.14. Aspectos éticos

A presente pesquisa atende aos postulados da Declaração de Helsinque emendada em Seul 2009, e segue os termos preconizados pelo Conselho Nacional de Saúde (Resolução 196 de 1996) para pesquisa em seres humanos. O projeto será submetido à

apreciação do Comitê de Ética em Pesquisa (CEP) do IMIP, após ser autorizado pela coordenação médica do CAM-IMIP e da Maternidade Petronila Campos.

Todas as pacientes serão devidamente informadas sobre os objetivos e métodos do estudo e só serão incluídas aquelas que concordarem em participar, assinando o Termo de Consentimento Livre e Esclarecido (TCLE) (Apêndice 2). Fica claramente resguardado o direito de qualquer paciente se recusar a participar do estudo, sendo assegurada a garantia de tratamento para todas, independente de sua participação.

A pesquisa terá início apenas depois da aprovação pelo Comitê de Ética em Pesquisa do IMIP e será interrompida na vigência de efeitos colaterais graves ou caso de demonstre na análise ínterim superioridade de um dos tratamentos.

Ressalta-se que o uso da drenagem placentária não é um procedimento padrão nos serviços envolvidos, uma vez que os profissionais que assistem às puérperas não seguem uma conduta padronizada no que diz respeito às alternativas usadas para reduzir o tempo do terceiro período e a perda sanguínea nesse período. Desta forma, alguns profissionais indicam o uso da drenagem placentária, por acreditarem particularmente em seus efeitos, e outros não.

De acordo com os resultados do presente estudo, poderá ser alterada a rotina da instituição, estabelecendo-se um protocolo para utilizar ou não a drenagem placentária em puérperas.

Os pesquisadores se comprometem a publicar os resultados do estudo em revistas indexadas, independente se forem favoráveis ou não à drenagem placentária.

5.14.1. Termo de Consentimento Livre e Esclarecido

Antes de ser incluída como amostra da pesquisa, a mulher será esclarecida, pelo pesquisador, a cerca dos objetivos, justificativa e metodologia do ensaio clínico com linguagem acessível e de fácil compreensão para a mesma.

Depois de esclarecidas todas as dúvidas, a possível participante será convidada a assinar o TCLE (Apêndice 2), que constará de todos os itens previstos pela Resolução 196/96 do Conselho Nacional de Saúde. Esse Termo será lido em voz alta para cada candidata a participar do estudo, sendo esclarecidas eventuais dúvidas durante a sua leitura.

5.14.2. Conflito de Interesses

Esta pesquisa está livre de conflitos de interesse particular ou institucional. Os equipamentos para a pesquisa serão adquiridos com recursos dos próprios pesquisadores.

VI. PLANO DE APRESENTAÇÃO DOS RESULTADOS

Os resultados serão apresentados em tabelas e/ou gráficos, dos quais fornecemos alguns exemplos:

Tabela 1. Características sócio-demográficas e obstétricas das mulheres submetidas ou não à drenagem placentária no terceiro período do parto, atendidas no setor de baixo risco do IMIP, de junho a novembro de 2012.

Características	Com drenagem placentária	Sem drenagem placentária
**Idade (X/DP)**		
**Escolaridade (X/DP)**		
**Renda per capta (X/DP)**		
**Gestações (X/DP)**		
**Paridade (X/DP)**		
**Raça (n/%)**		
Parda		
Branca		
Negra		
Indígena		
Amarela		
**IMC* (n/%)**		
Baixo peso		
Adequado		
Sobrepeso		
Obesidade		
**Procedência (n/%)**		
Recife e RMR		
Interior do estado		
Outro estado		
**Situação marital (n/%)**		
Com companheiro		
Sem companheiro		
**Tipos de parto anteriores (n/%)**		
Transvaginal		
Cesárea		
**Posição no parto (n/%)**		
Verticalizada		
Não verticalizada		
**Realização de episiotomia (n/%)**		
Sim		
Não		
**Laceração espontânea (n/%)**		
Sim		
Não		

* IMC
RMR: região metropolitana do Recife
n: número da amostra
X: média
DP: desvio padrão

Tabela 2. Desfechos maternos primários das parturientes atendidas no setor de baixo risco do IMIP submetidas ou não à drenagem placentária, no período de junho a novembro de 2012. Recife, PE, 2012.

Desfechos maternos primários	Com Drenagem Placentária		Sem Drenagem Placentária		*p* *
	Média + DP	Variação	Média + DP	Variação	
Duração do terceiro período (minutos)					
Perda sanguínea (ml)					
Níveis de hematócrito antes do parto (%)					
Níveis de hematócrito 24-48 h após o parto (%)					
Perda sanguínea estimada (ml): método de Wintrobe					

DP: desvio padrão, * Teste t de *Student*

Tabela 3. Desfechos maternos secundários das parturientes atendidas no setor de baixo risco do IMIP submetidas ou não à drenagem placentária, no período de junho a novembro

**Desfechos maternos secundários**	**Com drenagem placentária**		**Sem drenagem placentária**		RR	IC 95%	*p*
	N	%	N	%			
**HPP > 500 ml**							
**HPP > 1000 ml**							
**Hemoglobina materna < 8g/dl 24-48 h pós-parto**							
**Hemotransfusão após o parto**							
**Dor abdominal no puerpério**							
**Uso terapêutico de ocitócitos na primeira hora pós-parto**							
**Uso terapêutico de ocitócitos dentro de até 24h pós-parto**							
**Terceiro período > 30 minutos**							
**Terceiro período > 60 minutos**							
**Curagem uterina**							
**Remoção manual da placenta**							
**Curetagem uterina**							
**Sintomas de anemia até 48 h pós-parto**							
**Satisfação materna: sim corresponde às categorias 0 e 1 da figura 02**							

de 2012. Recife, PE, 2012.

HPP: hemorragia pós-parto; RR: risco relativo, IC: intervalo de confiança, N: amostra, %: percentagem, * Teste chi-quadrado

VII. CRONOGRAMA

MESES / ETAPAS	1*	2	3	4	5	6	7	8	9	10	11	12	13	14	15	16	17	18	19	20	21	22
Elaboração do projeto	X	X	X	X	X	X	X	X														
Qualificação do projeto									X													
Comitê de Ética										X	X											
Revisão da literatura	X	X	X	X	X	X	X	X	X	X	X	X	X	X	X	X	X	X	X			
Coleta dos dados											X	X	X	X	X	X						
Revisão e correção dos formulários preenchidos												X	X	X	X	X						
Digitação												X	X	X	X	X	X					
Revisão da digitação, limpeza e testes de consistência																	X					
Tabulação e análise dos dados																	X	X				
Revisão da análise dos dados																	X	X				
Redação do artigo e da dissertação																		X	X			
Revisão da dissertação e preparação para publicação																				X		
Envio para publicação																					X	
Defesa da dissertação																						X

* O mês "1" corresponde ao mês de julho do ano de 2011.

VIII. ORÇAMENTO

Item	Quantidade	Preço		Justificativa
		Unitário	Total	
**1. Capital**				
**1.1 Equipamento e material permanente**				
Balança digital	01	500,00	500,00	Pesar os sacos plásticos que contêm o sangue drenado
Mouse	01	40,00	40,00	Digitação de banco de dados e impressão de Formulários e pesquisa
Pen drive	01	50,00	50,00	
Grampeador	01	30,00	30,00	
**1.2 Material Bibliográfico**				
Solicitação de artigos científicos	30	10,00	300,00	Escrever a discussão
**Subtotal material permanente**			**920,00**	
**2. Custeio**				
**2.1 Serviços de terceiros**				
Estatístico	01	1.500,00	1.500,00	Análise de dados
Tradutor (por lauda)	20	40,00	800,00	Para publicação em revista internacional
**2.2 Material para medição volêmica**				
Sacos plásticos	284	0,14	39,76	Intervenção
**2.3 Material laboratorial**				
Hematócrito antes do parto	226	1,53	345,78	Intervenção
Hemoglobina antes do parto	226	1,53	345,78	Intervenção
Hematócrito 24-48 h após o parto	226	1,53	345,78	Intervenção
Hemoglobina 24-48 h após o parto	226	1,53	345,78	Intervenção
**2.4 Material de consumo**				

Papel A4 (resma)	2	15,00	30,00	Impressão de formulários, documentos e pesquisa
Cartucho preto	2	30,00	60,00	
Cartucho colorido	2	30,00	60,00	
CD RW	10	2,5	25,00	Coleta de dados
Canetas	10	0,70	7,00	
Grampos (caixa)	01	20,00	20,00	
Pasta Arquivos/colecionador	10	3,00	30,00	
Prancheta	02	14,00	28,00	
Calculadora	01	15,00	15,00	
Etiquetas rolos	02	1,50	3,00	Marcação dos prontuários
**Subtotal custeio+capital**			**4.642,88**	
**Total (custeio + capital+material permanente)**			**4.920,88**	

- **A pesquisadora já disponibiliza de notebook próprio e impressora para a realização da pesquisa;**
- **Total de sacos plásticos: 284 unidades, já incluídos 20% do total da amostra (226) por possíveis perdas;**

IX. REFERÊNCIAS

1. Prendiville Walter JP, Elbourne Diana, Susan J. McDonald Active Versus Expectant Management in The Third Stage of Labour. Cochrane Database of Systematic Reviews. In: *The Cochrane Library*, Issue 04, art. 2009.
2. Clinical guidelines. Intrapartum care: care of healthy women and their babies during childbirth. National Collaborating Centre for Women's and Childrens's Health. London: RCOG Press; 2007.
3. WHO/UNICEF (World Health Organization/ United Nations International Children's Emergency Fund), 2005. Postpartum hemorrhage, manual for teachers of midwifery. Disponível em: http://whqlibdoc.who.int/publications/2005/9248546668_2_por.pdf
4. Khan K. WHO analysis of causes of maternal death: a systematic review. The Lancet. Abril de 2006, Pages 1066-1074.
5. Ghazal-Aswad S , P Badrinath , Sidky Eu , Abdul Razak-Y , Davison J , Mirghani HM .Confidential enquiries into maternal mortality in the United Arab Emirates: a feasibility study. J Obstet Gynaecol Res. 2011 Mar; 37 (3) :209-14.
6. WHO/UNICEF (World Health Organization/ United Nations International Children's Emergency Fund), Recommendations for the Prevention of postpartum hemorrhage.2007http://whqlibdoc.who.int/hq/2007/WHO_MPS_07.06_eng.pdf
7. WHO/UNICEF (World Health Organization/ United Nations International Children's Emergency Fund), 1996. Postpartum hemorrhage, manual for teachers of midwifery. Disponível em: http://whqlibdoc.who.int/publications/2005/9248546668_2_por.pdf
8. Begley Cecília M, Gyte Gillian ML, Deirdre Murphy J, Devane Declan, McDonald Susan J, William McGuire. Active Versus Expectant Management in

The Third Stage of Labour. Cochrane Database of Systematic Reviews. In: *The Cochrane Library,* Issue 04, art. 2011.

9. Joseph KS, Rouleau J, Kramer MS, Young DC, RM Liston, Baskett TF. Investigation of an increase in postpartum haemorrhage in Canada. *BJOG.* 2007; 114 (6):751759. doi:10.1111/j.14710528.2007.01316.x
10. Lain SJ, CL Roberts, RM Hadfield, Bell JC, JM Morris. Como é a precisão do relato de hemorragia obstétrica de dados a alta hospitalar? Um estudo de validação. *Aust NZJ Gynaecol Obstet.* 2008; 48 (5). :481-484.
11. McDonald Susan J, Abbott Jo M, Higgins Shane P. Prophylactic Ergometrine-Oxytocin Versus Oxytocin For The Third Stage of Labour. Cochrane Database of Systematic Reviews. In: *The Cochrane Library,* Issue 04, art. 2009
12. Cavaleiro M , WM Callaghan , Berg C , Alexander S , Bouvier-Colle MH , Ford JB , Joseph KS , Lewis G , RM Liston , Roberts CL , aveia J , J Walker .Trends in postpartum hemorrhage in high resource countries: a review and recommendations from the International Postpartum Hemorrhage Collaborative Group. BMC Pregnancy Childbirth. 2009 Nov 27.
13. Gogarten W . Postpartum hemorrhage - an update. Anasthesiol Intensivmed Schmerzther Notfallmed. 2011 Jul; 46 (7-8) :508-14.
14. Cameron CA , CL Roberts , Olive CE , Ford JB , Fischer WE .Trends in postpartum haemorrhage. Aust Saúde Pública NZJ. 2006 Apr; 30 (2) :151-6 .
15. Mutihir JT , Utoo BT. Postpartum maternal morbidity in Jos, North Central Nigeria. Niger J Clin Pract. 2011 Jan-Mar; 14 (1) :38-42.
16. Costa Aurélio Antônio Ribeiro, Ribas Maria do Socorro Sampaio de Sousa, Amorim Melania Maria Ramos de, Santos Luiz Carlos. Mortalidade materna na cidade do Recife. Rev. Bras. Ginecol. Obstet. [serial on the Internet]. 2002 Aug.
17. Kramer MS , Dahhou M , Vallerand D , R Liston , Joseph KS . Risk factors for postpartum hemorrhage: can we explain the recent temporal increase? J Obstet Gynaecol Can. 2011 Ago; 33 (8) :810-9.
18. Blomberg M. Maternal obesity and risk of postpartum hemorrhage.Obstet Gynecol 2011 Sep; 118 (3) :561-8.
19. C. Le Ray, W. Fraser, P. Rozenberg, B. Langer, D. Subtil, F. Goffinet. Duration of passive and active phases of the second stage of labour and risk of severe

postpartum haemorrhage in low-risk nulliparous women. European Journal of Obstetrics & Gynecology and Reproductive Biology2 June 2011.

20. Rossen J , I Okland , Nilsen OB , Eggebo TM . Is there an increase of postpartum hemorrhage, and is severe hemorrhage associated with more frequent use of obstetric interventions? . Acta Obstet Gynecol Scand 2010 Oct; 89 (10) :1248-55.
21. Combs CA, Murphy EL, Laros RK Jr. Factors associated with postpartum hemorrhage with vaginal birth. *Obstet Gynecol.* 1991; 77 (1). :69-76
22. Bateman BT , Berman MF , Riley LE , Leffert LR .The epidemiology of postpartum hemorrhage in a large, nationwide sample of deliveries. Anesth Analg. 2010 01 de maio; 110 (5) :1368-73.
23. Lars Hoj, Placido Cardoso, Birgitte Bruun Nielsen, Lone Hvidman, Jens Nielsen, Peter Aaby. Effect of sublingual misoprostol on severe postpartum haemorrhage in a primary health centre in Guinea-Bissau: randomised double blind clinical trial. BMJ. 2005 01; 331 (de outubro 7519) : 723
24. Jongkolsiri P, Manotaya S. Placental cord drainage and the effect on the duration of third stage labour, a randomized controlled trial. Cochrane Database of Systematic Reviews. In: *The Cochrane Library,* Issue 04, art. 2009.
25. Soltani Hora, Poulose Thomas A, Hutchon David R. Placental cord drainage after vaginal delivery as part of the management of the third stage of labour. Cochrane Database of Systematic Reviews. In: *The Cochrane Library,* Issue 01, Art. No. CD004665. DOI: 10.1002/14651858.CD004665.pub4. 2011.
26. Hossain N , T Shah , Khan N , N Shah , Khan NH .Transfusion of blood and blood component therapy for postpartum haemorrhage at a tertiary referral center. J Med Assoc Pak 2011 Apr; 61 (4) :343-5.
27. Brasil. Ministério da Saúde. Parto, Aborto e Puerpério. Assistência Humanizada à Mulher. Brasília, 2001.p. 77-85
28. Rebecca Lynn Coelius, Amy Stenson, Jessica L. Morris, Mingji Cuomu, Carrie Tudor,Suellen Miller. The tibetan uterotonic zhi byed 11: mechanisms of action, efficacy, and historical use forpostpartum hemorrhage. Baseada Evid Complemento alternando Med. 2012; 2012: 794164. Publicados on-line 24 julho 2011.
29. Lars Hoj, Placido Cardoso, Birgitte Bruun Nielsen, Lone Hvidman, Jens Nielsen, Peter Aaby. Effect of sublingual misoprostol on severe postpartum

haemorrhage in a primary health centre in Guinea-Bissau: randomised double blind clinical trial. BMJ. 2005 01; 331 (de outubro 7519) : 723.

30. Liabsuetrakul Tippawan, Choobun Thanapan, Peeyananjarassri Krantarat, Islam Q Monir. Prophylactic use of ergot alkaloids in the third stage of labour. Cochrane Database of Systematic Reviews. In: *The Cochrane Library,* Issue 04, Art.2009.
31. Cotter Amanda M, Ness Amen, Tolosa Jorge E.Prophylactic Oxytocin for the Third Stage of Labour. Cochrane Database of Systematic Reviews.In: *The Cochrane Library,* Issue 04, art. 2009.
32. Arias F. Pharmacology of oxytocin and prostaglandins. Clin Obstet Gynecol. 2000; 43:455-68.
33. Nasreen HE , Nahar S , Al Mamun M , Afsana K , P ByassRisk factors for postpartum hemorrhage: can we explain the recent temporal increase? Ação Glob Health. 2011; 4. doi: 10.3402/gha.v4i0.7017.
34. Hofmeyr GJ , Fawole B , Mugerwa K , NP Godi , Blignaut Q , Mangesi L , M Singata , Brady L , J Blum . Administration of 400 µg of misoprostol to augment routine active management of the third stage of labor. Int J Gynaecol Obstet 2011 Fev; 112 (2) :98-102.
35. Gülmezoglu A, Forna, Villar J, Hofmeyr G Justus. Prostaglandins for preventing postpartum hemorrhage. Cochrane Database of Systematic Reviews. In: *The Cochrane Library,* Issue 04, art. 2011.
36. Gülmezoglu *et al.* Active management of the third stage of labour with and without controlled cord traction: a randomised, controlled, non-inferiority Trial. Lancet. March, 2012. DOI:10.106/S0140-6736(12)60206-2.
37. Rabe H, Reynolds GJ, Diaz-Rosello JL. Early versus delayed umbilical cord clamping in preterm infants. The Cochrane Library, Issue 1, 2010.
38. Ceriani Cernadas JM , Carroli G , L Pellegrini , Ferreira M , C Ricci , Casas O , Lardizabal J , Morasso mdel C .The effect of early and delayed umbilical cord clamping on ferritin levels in term infants at six months of life: a randomized, controlled trial. Arch Argent Pediatr 2010 Jun; 108 (3) :201-8.
39. Leduc D , Senikas V , Lalonde AB , C Ballerman , Biringer A , Delaney M , L Duperron , Girard I , D Jones , Lee LS , Pastor D ,Wilson K. Active management

of the third stage of labour: prevention and treatment of postpartum hemorrhage. J Obstet Gynaecol Can. 2009 Out; 31 (10) :980-93.

40. Peña-Martí Guiomar E, Comunián-Carrasco Gabriella. Fundal pressure versus controlled cord traction as Part of The Active Management of Third Stage of Labour. Cochrane Database of Systematic Reviews. In: *The Cochrane Library*, Issue 04, art. 2009.
41. Hofmeyr G Justus, Abdel-Aleem Hany, Mahmoud Abdel-Aleem massagem A. uterina para prevenção de hemorragia pós-parto.Cochrane Database of Systematic Reviews. In: *The Cochrane Library*, Issue 08, art 2011.
42. Althabe F , A Alemán , Tomasso G , L Gibbons , Vitureira G , Belizán JM , Buekens P . A pilot randomized clinical trial of controlled cord traction to reduce blood loss after delivery.Int J Gynaecol Obstet 2009 Out; 107 (1) :4-7.
43. Giacalone PL, Vignal J, Daures JP, Boulot P, Hedon B, Laffargue F. A randomised evaluation of two techniques of management of the third stage of labour in women at low risk of postpartum haemorrhage. BJOG. 2000 Mar;107(3):396-400.
44. Sharma JB, Pundir P, Malhotra M, Arora R. Evaluation of placental drainage as a method of placental delivery in vaginal deliveries. Arch Gynecol Obstet. 2005 Apr;271(4):343-5. Epub 2004 Mar 18
45. L.M.; Furdeg, C.D.; De Mates, D. L. Sample size. In: Clinical Train. 3rd. Editora Mosby, 1996. Cap. 07, pp. 94-129.
46. Moher D, Schultz KF, Altman DG. The CONSORT statement: revised recommendations for improving the quality of reports of parallel-group randomized trials. Lancet 2001; 357:1191-4.
47. Melo MIB, Mello MG. Assistência de enfermagem ao trabalho de parto e parto de baixo risco. In: Santos *et al* . Enfermagem em ginecologia e obstetrícia. 1 edição. Rio de Janeiro: Medbook; 2010. p 99-115.
48. Filho, RM. Parto. Estudo clínico e assistência. In:______ Obstetrícia fundamental. 11 ed. Rio de Janeiro: Guanabara Koogan, 2008. p 164-175.
49. Filho, RM. Rotura uterina. Laceração de trajeto. In:______ Obstetrícia fundamental. 11 ed. Rio de Janeiro: Guanabara Koogan, 2008. p 463-470.
50. Oliveira L *et al.* Cálculo da estimativa d perda sanguínea. 1985.

51. Collins, S. L.; Moore, R. A.; McQuay, H. J., The visual analogue pain intensity scale; what is moderate pain in millimeters? **Rev Pain**, 72: 95-97, 1997.

APÊNDICE 1

LISTA DE CHECAGEM

"EFETIVIDADE DA DRENAGEM PLACENTÁRIA PARA REDUÇÃO DA DURAÇÃO DO TERCEIRO ESTÁGIO DO PARTO E DA PERDA SANGÜÍNEA PÓS-PARTO: ENSAIO CLÍNICO RANDOMIZADO"

Paciente nº. ☐☐☐

Formulário nº. ☐☐☐

Pesquisador: ______________________________

Data: ☐☐/☐☐/☐☐☐☐

IDENTIFICAÇÃO

Nome: ____ Registro: ☐☐☐☐☐☐☐☐

Idade: ☐☐ anos

CRITÉRIOS DE INCLUSÃO

Parturientes de baixo risco obstétrico	☐ **SIM**	☐ **NÃO**
Gestação a termo (37 a 42 semanas)	☐ **SIM**	☐ **NÃO**
Parto assistido no setor de baixo risco	☐ **SIM**	☐ **NÃO**
Feto vivo	☐ **SIM**	☐ **NÃO**

CRITÉRIOS DE EXCLUSÃO

Mulheres incapazes	☐ **SIM**	☐ **NÃO**
Mulheres que concordaram em participar, assinaram o TCLE, porém evoluíram para cesariana ou fórceps	☐ **SIM**	☐ **NÃO**

☐ INCLUÍDA ☐ EXCLUÍDA

Aceitou participar da pesquisa: ☐ SIM ☐ NÃO

APÊNDICE 2

TERMO DE CONSENTIMENTO LIVRE E ESCLARESCIDO

(De acordo com os critérios da resolução 196/96 do Conselho Nacional de Saúde)

Cara senhora, você está sendo convidada como voluntária a participar da pesquisa:

"EFETIVIDADE DA DRENAGEM PLACENTÁRIA NO TERCEIRO PERÍODO DO PARTO: ENSAIO CLÍNICO RANDOMIZADO"

A JUSTIFICATIVA, OS OBJETIVOS E OS PROCEDIMENTOS

O motivo que nos leva a estudar o problema é que quando se realiza a retirada da pinça que prende o cordão umbilical, haverá redução no tempo de saída da placenta e do risco de sangramento excessivo, porém precisamos saber qual a quantidade de sangue que perdemos nesse momento e escolher qual a melhor forma de agir nessa hora. Por isso, surgiu a necessidade de se realizar esta pesquisa.

Nesse estudo realizaremos ou não a retirada da pinça que prende o cordão umbilical depois que o bebê nascer. Apenas iremos comparar duas situações: a primeira será a retirada da pinça que prende o cordão umbilical quando o bebê nasce e a outra a pinça não será retirada. Para isso haverá uma sorteio para definir se a senhora será ou não retirada a pinça.

DESCONFORTOS, RISCOS E BENEFÍCIOS

A realização ou não da retirada da pinça que prende o cordão umbilical placentária não ocasionará efeitos colaterais na senhora, nem comprometerá o bem estar do seu bebê. Além do que se faz de rotina, será feita a coleta de sangue da senhora para avaliar anemia

uma vez antes e uma vez depois do parto. Poderá acontecer um pequeno desconforto (como dor local, discreta irritação ou ficar arroxeado) quando for retirar sangue apenas da senhora antes e 24 horas após o parto para verificar se existe anemia. Essa técnica tem um risco mínimo, o mesmo de qualquer exame de sangue.

FORMA DE ACOMPANHAMENTO E ASSISTÊNCIA

A senhora será acompanhada por uma equipe formada por enfermeiros e médicos obstetras, neonatologistas e técnicas de enfermagem. Nesse setor, os enfermeiros que prestam a assistência são obstetras juntamente com os residentes de enfermagem que estão se especializando em saúde da mulher. Esses profissionais são devidamente capacitados para cuidar da senhora e caso haja necessidade, o médico obstetra será chamado para avaliar a senhora.

Durante a realização da pesquisa (acompanhamento) não haverá alteração da conduta habitualmente utilizada no IMIP.

GARANTIA DE ESCLARECIMENTO, LIBERDADE DE RECUSA E GARANTIA DE SIGILO

A senhora será esclarecida sobre o que desejar na pesquisa e é livre para não participar se não quiser e sair a qualquer momento. A sua participação é voluntária e a vontade de não participar não lhe trará problemas, ou seja, mesmo que a senhora não queira participar, continuará sendo atendida da mesma forma pelos profissionais do hospital.

O(s) pesquisador(es) se comprometem em não dizer seu nome ou divulgar o material que mostre sua participação na pesquisa. A senhora não será identificada em nenhum momento. Uma cópia deste consentimento informado será arquivada no seu prontuário.

CUSTOS DA PARTICIPAÇÃO

A participação no estudo não acarretará custos para a senhora. Todos os exames serão realizados pelo IMIP.

DECLARAÇÃO DA PARTICIPANTE

Eu, ____ paciente matriculada no IMIP com o registro □□□□□□□□, declaro que fui informada dos objetivos e finalidade da pesquisa **"Efetividade da drenagem placentária no terceiro período do parto: ensaio clínico randomizado**" de maneira clara e detalhada e esclareci minhas dúvidas. Sei que em qualquer momento poderei solicitar novas informações e alterar a minha decisão, sem que isso venha a prejudicar meu atendimento no IMIP. O(a) pesquisador(a) ________________________________ certificou-me de que todos os dados desta pesquisa serão confidenciais. Também sei que caso existam gastos adicionais, estes serão absorvidos pelo pesquisador responsável. Em caso de dúvidas poderei chamar o pesquisador __________________________ no telefone (81) 8782-8552 ou contactar o Comitê de Ética em Pesquisa do IMIP, número (81) 2122-4706. Declaro ainda que concordei em seguir todas as orientações do pesquisador, que concordei em participar desse estudo, que recebi uma cópia deste termo de consentimento livre e esclarecido e me foi dada a oportunidade de ler e esclarecer as minhas dúvidas.

____

Nome Assinatura do Participante Data

____

Nome Assinatura do Pesquisador Data

____

Nome Assinatura da Testemunha Data

Informações sobre o andamento da pesquisa podem ser encontradas no site do Sistema Nacional de Ética em Pesquisa (SISNEP): http://portal.saude.gov.br/sisnep/pesquisador/menuusuario.cfm

APÊNDICE 03

Efetividade da Drenagem Placentária

Número do formulário: □□□
Número de registro: □□□□□□
Data da coleta: □□/□□/□□□□
Pesquisador: ________________

Primeira Revisão: □□/□□/□□□□ Revisor:_________________
Segunda Revisão: □□/□□/□□□□ Revisor:_________________

Identificação:
Nome:____
Data de nascimento: □□/□□/□□□□

Variáveis Biológicas

Idade(anos)	□ □
Raça	1. □ Branca 2. □ Negra 3. □ Amarela 4. □ Indígena<br>5. □Parda
Peso	□□□□
Altura	□,□□
IMC	□□

Variáveis Sociodemográficas

Procedência	1. □ Recife e RMR 2. □ Interior do estado<br>3. □ Outro estado
Escolaridade (anos)	□□
Situação marital	1. □ Com companheiro 2. □ Sem companheiro
Renda familiar total per capta	□□□□□□ reais
Número de pessoas	□□

Variáveis Obstétricas

Número de gestações (incluindo a atual) □□

Paridade □□

Tipos de parto anteriores 1. □ Transvaginal 2. □ Cesárea

Características desse parto

Posição no parto 1. □ Verticalizada 2. □ Não verticalizada

Realização de episiotomia 1. □ Sim 2. □ Não

Lacerações espontâneas 1. □ Presente 2. □ Ausente

Desfechos maternos primários

Duração de terceiro período □□□ minutos

Perda sanguínea □□□□ ml

Níveis de hematócrito antes do parto □□ %

Níveis de hematócrito 24 h após o parto □□ %

Desfechos maternos secundários

HPP > 500 ml na primeira hora 1. □ Sim 2. □ Não

HPP > 1000 ml na primeira hora 1. □ Sim 2. □ Não

Hemoglobina materna < 8g/dl 24-48 h pós-parto 1. □□

Hemotransfusão após o parto 1. □ Sim 2. □ Não

Dor abdominal no puerpério 1. □ Sim 2. □ Não

1.□Sim 2. □ Não

Uso terapêutico de ocitócitos na primeira hora pós-parto

Uso terapêutico de ocitócitos dentro de até 24h pós-parto 1. □ Sim 2. □ Não

Terceiro período > 30 min 1. □ Sim 2. □ Não

Terceiro período > 60 min 1. □ Sim 2. □ Não

Curagem uterina 1. □ Sim 2. □ Não

Remoção manual da placenta 1. □ Sim 2. □ Não

Curetagem uterina 1. □ Sim 2. □ Não

Sintomas de anemia até 48 h até pós-parto (tontura, cefaleia, cansaço)	1. ☐ Sim 2. ☐ Não
Satisfação materna	0. ☐ Muito satisfeita 1. ☐ Satisfeita<br>2. ☐ Pouco satisfeita 3. ☐ Insatisfeita<br>4. ☐ Muito insatisfeita

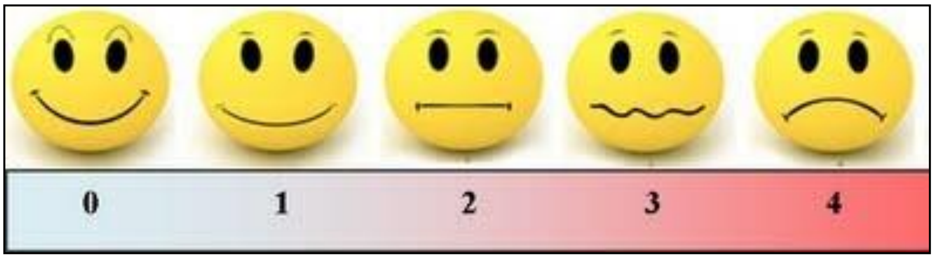

Figura 2: Escala de faces para avaliação da satisfação maternal com o manejo do terceiro período

APÊNDICE 4

CARTA DE ANUÊNCIA

Declaro, para os devidos fins, que concordo em participar do Projeto de Pesquisa, intitulado: Efetividade da drenagem placentária no terceiro período do parto: ensaio clínico randomizado, sob a responsabilidade de Fernanda Barros Lima Vasconcelos mestranda em Saúde Materno Infantil**,** do Instituto de Medicina Integral Professor Fernando Figueira, desenvolvendo as atividades de que competem à pesquisadora de análise de dados do setor de baixo risco da IMIP, através da aplicação da drenagem placentária nas pacientes atendidas no setor. Com base nessas informações, a coleta de dados será feita diretamente com as pacientes.

____

Leila Katz coordenadora do PPP do IMIP

____

Fernanda Barros Lima Vasconcelos
Identidade: 4515677 SDS/PE
CPF: 02758647400
Fone(s) para contato: (081) 34450448/ 87828552
E-mail: barrosvida@yahoo.com.br
