# Supplementary material for: Placental cord drainage in the third stage of labor: Randomized clinical trial
Source: PLoS One. 2018 May 2;13(5):e0195650. doi: 10.1371/journal.pone.0195650 (PMC5931461; doi:10.1371/journal.pone.0195650)
Supplement: S2 File — (PDF) [file pone.0195650.s002.pdf]

V. METHODS

5.1. Study Design

A randomized open-label trial will be conducted comparing women in the third stage of labor who will undergo placental drainage or not.

5.2. Place of study

The study will be conducted at IMIP and Petronila Campos Hospital where low-risk women are assisted during labor / delivery and puerperium, where low-risk obstetric women are attended during pre-delivery, delivery and postpartum (LDR).

5.3. Period of data collection

The data will be collected in the period between June and November 2012.

5.4. Study population It includes all low-risk, full-term obstetric women with a live fetus in labor treated in the low-risk sectors during pre-delivery / delivery and puerperium.

5.5. Sample

5.5.1. Sampling A non-probabilistic sample of convenience will be obtained, composed of the pregnant women attended during the study period who meet the eligibility criteria. Patients eligible for the study will be randomized into two groups: with and without placental drainage.

5.5.2. Sample size The sample size was calculated using the following formula for dichotomous variables:

$$ZN = \frac{4(Z\alpha + Z\beta)^2\,\bar{p}(1 - \bar{p})}{(p_c - p_I)^2}$$

At where:

ZN = total sample number (N = number of participants per group)

$Z\alpha$ = alpha error Z score (1.96 for an alpha error of 5%)

$Z\beta$ = Z-score of the beta error (1.282 for a power of 90%)

p = (pc + pI) / 2

p_c = proporção no grupo do controle

p_I = proporção no grupo de intervenção.

The OpenEpi version 2.3 (Atlanta, GA), in the public domain, was used for this calculation. The parameter used to calculate the sample size was the duration of the third period of labor. A randomized clinical trial published in 2009 showed that there is reduction in the third period of labor with placental drainage (5.1 ± 2.4 minutes versus 7.0 ± 6.1 minutes) 24.

Considering a level of significance of 5% and a power of 80%, it would be necessary 188 women to show difference between the groups. Predicting eventual losses, this number was increased to 226 postpartum women, of whom 113 were in each group.

5.6. Criteria and procedures for selecting participants

5.6.1. Inclusion criteria

• Parturients with low obstetric risk;

• Single term pregnancy (37 to 42 weeks);

• assisted delivery in the services where the study will be conducted, where low-risk women are assisted during labor / delivery and puerperium;

• Live fetus.

5.6.2. Exclusion Criteria

• Women with disabilities;

• Women who agreed to participate, signed the ICF, but did not progress to cesarean section and forceps.

5.6.3. Procedures for Funding and Follow-up of Participants

Before starting the data collection, a sensitization of all the professionals will be carried out, promoting the dissemination of the project by the responsible researchers, through posters and oral communication, explaining the objectives, justification and the methods of the research. These professionals will be previously trained by the researchers.

These professionals will identify the possible women candidates for the study. Once a potential participant is identified, the researcher / assistant will apply a checklist (Appendix 1) to confirm compliance with the eligibility criteria. If the pregnant woman fulfills the criteria, she will be approached by the researcher / assistant, and will receive information about the reasons for the research, and its importance, being invited to join this one. The pregnant women will also be informed that if they do not agree to participate in the study, their follow-up will be performed according to the usual routine of the service by a qualified team, without any detriment of any order. If you voluntarily agree to participate, you will be asked to sign the Informed Consent Form (Appendix 2), after careful reading and explanations by the researcher / assistant. Subsequently, a blood sample will be collected by the laboratory professional before delivery and referred for laboratory analysis. All participants will be assisted in labor performed according to the routine of the service and immediately after the delivery, the sealed envelopes containing the group to which each participant will be allocated will be opened (Figure 1). The collection will be performed by researchers, obstetrical nurses of the sector during the day, night and weekends. These professionals will be trained prior to the start of the research. After 24-48 h a new blood sample will be collected by the laboratory employee and referred for laboratory analysis.

5.7. Flowchart for assessment and following participants

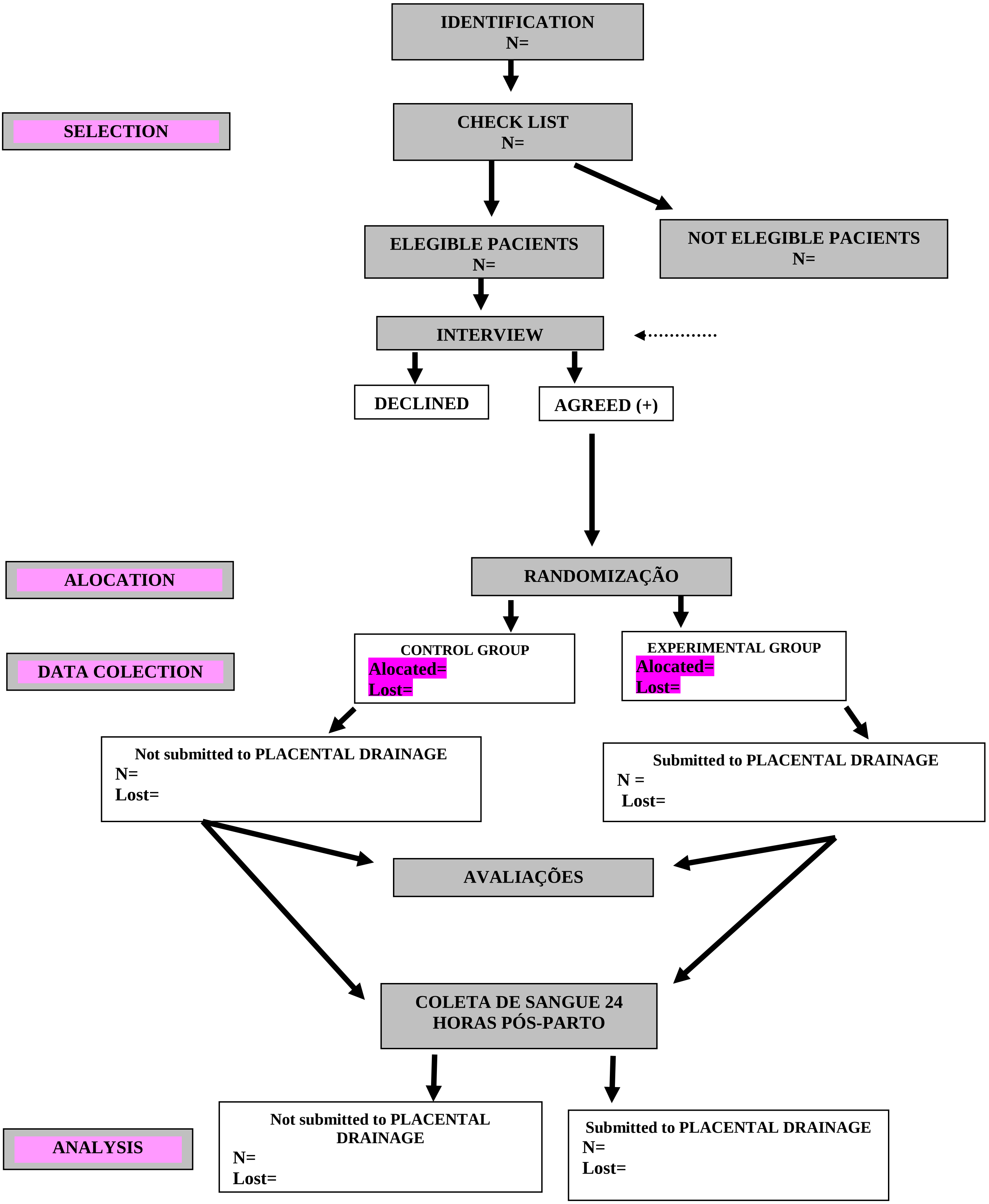

Figure 1. Flowchart of abstraction and follow-up of participants (CONSORT flowchart).

5.7.1. Procedures for randomization Randomization for placental drainage or not will be done according to a randomly generated list of random numbers using the Random Allocation Software version 1.0 program. From this list, envelopes numbered from 1 to 226 will be prepared, and each number, according to the randomization list, will correspond to the allocation of the patient in the experimental or control (GE or GC) group. These envelopes will be prepared by a project partner, who will not be part of the data collection. The patient will be invited to participate in the study and if she agrees, she will sign the Free and Informed Consent Form (Appendix 2). After delivery, those who have agreed and signed the ICF and who have evolved with spontaneous vaginal delivery will receive the sealed and numbered envelope containing information about their allocation, which is attached to the medical record. Only then will the opening of the sealed envelope containing the group selected for that participant be performed, guaranteeing the concealment of the allocation. The groups will be classified as follows: GE (experimental group): submitted to placental drainage GC (control group): not submitted to placental drainage.

Women who agree to pareticipate and who progress to cesarean section and forceps delivery will be excluded from the study, thus not receiving the sealed and numbered envelope.

5.8. Analysis variables:

5.8.1. Independent Variable:

• Placental drainage: yes or no

5.8.2. Dependent Variables:

5.8.2.1. Primary Outcomes:

• Duration of the third delivery period;

• Volume of blood loss one hour postpartum

5.8.2.2. Secondary Outcomes:

• Postpartum haemorrhage (> 500ml) in the first hour

• Severe postpartum haemorrhage (> 1000ml) in the first hour;

• Maternal hemoglobin with 24-48 postpartum less than 8g / dl;

• Difference in hematocrit (pre and 24-48 h postpartum);

• Need for blood transfusion;

• Abdominal pain in the puerperium;

• Need for therapeutic use of oocytes in the first hour postpartum;

• Need for therapeutic use of oocytes within 24 h postpartum

• Third period greater than 30 minutes;

• Third period greater than 60 minutes;

• Need for uterine healing;

• Manual removal of the placenta;

• Need for uterine curettage;

• Symptoms of anemia up to 48 hours postpartum (dizziness, headache, tiredness);

• Maternal satisfaction with the management of the third period.

5.9. Definition and operation of variables

5.9.1. Terms

• Third period of delivery - the period between the birth of the baby and the expulsion of the placenta1.

• Placental drainage - consists of the removal of the umbilical cord, which was fixed during delivery to separate it from the placenta to allow blood drainage25.

5.9.2. Inclusion criteria

• Low-risk parturients - the onset of spontaneous labor in the term, with no identified risk factor, remaining in this condition throughout the labor process.

• Full-term pregnancy - gestations between the 37th and 42nd week of single-fetus pregnancy, calculated from the date of the last menstrual period (DUM), since known and reliable, confirmed by the first ultrasound examination. When the patient does not know the DUM and / or disagrees with the ultrasonographic examination, the gestation will be performed based on the first ultrasound.

• Low-risk women assisted during labor / delivery and puerperium.

• Live fetus - positive fetal auscultation on admission determined by the Pinard stethoscope or Doppler, and confirmed by ultrasound when auscultation is initially negative.

5.9.3. Exclusion Criteria

• Unable to consent: mentally retarded and indigenous;

• Women who agreed to participate, signed the ICF, but progressed with cesarean delivery or instrumental delivery to forceps;

• Instrumental delivery to forceps - labor assisted by the use of a metallic instrument, similar to a forceps, consisting of two articulated spoon-shaped arms, which will hold the baby's head and pull it out of the vaginal canal;

• C-section - is the surgical act that consists of incising the abdomen and the wall of the pregnant uterus to release the concept developed there.

5.9.4. Study Variables:

5.9.4.1. Descriptive variables

• Maternal age (years) - continuous numerical variable (which can later be categorized), expressed in complete years, according to the patient's information, at the time of her selection to enter the study.

• Maternal height (meters) - continuous numerical variable (which can later be categorized), expressed in meters, indicating its height. Information will be collected by the researcher / assistant or in the medical record, at the time of his / her selection to enter the study.

• Weight (kilos) - a continuous numerical variable (which can later be categorized), expressed in kilos, indicating the weight of the patient at the time of the study. Information will be collected by the researcher / assistant or on the patient's chart. • Body Mass Index (BMI) - BMI is calculated from height in meters and weight, using the formula: weight / height2. Continuous numerical variable, which can be later categorized for analysis purposes: low weight, weight adequate, overweight and obese, adopting the cut levels proposed by Atalah et al (APPENDIX 1).

• Race - polycotomic categorical variable. Considering that the study is prospective and the researcher can talk to women, they will define their race.

• Origin - a categorical polytomic variable, indicating the place where the woman resides, being: Recife and metropolitan region, interior of Pernambuco or other states. According to patient information.

• Schooling - discrete numerical variable, representing the level of schooling of the woman or puerperal, expressed in complete and approved years of study. It can be categorized later for analysis. According to patient information.

• Marital status - dichotomous categorical variable, expressing the presence or absence of the partner. According to patient information.

• Per capita family income - continuous numerical variable (which can later be categorized for analysis) revealing, in reais, the income per person in the family in which the mother is inserted. According to patient information.

• Number of pregnancies - discrete numerical variable, corresponding to the number of pregnancies, including the current one, having come to term or not. According to patient information.

• Parity - discrete numerical variable, corresponding to the number of previous deliveries completed, according to the patient's information.

• Previous delivery - dichotomous categorical variable, type yes or no, describing the presence or absence of previous delivery. According to patient information.

• Previous types of delivery - numerical variable, corresponding to the number of previous transvaginal deliveries and number of cesareans. According to patient information.

• Posture during delivery - dichotomous categorical variable, determining the positioning adopted by the woman at the time of delivery. They will be considered vertical or non-vertical position. As noted by the researcher / assistant.

• Episiotomy - a dichotomous nominal variable that expresses whether episiotomy is

performed or not. Collected in the medical record or according to the observation of the researcher / assistant. It is an incision made in the region of the perineum (muscle area between the vagina and the anus) to enlarge the birth canal and prevent irregular laceration during the baby's passage.

• Spontaneous laceration - dichotomous categorical variable, of the yes or no type, describing if there was any type of perineal laceration that occurred spontaneously at the time of delivery. Collected in the medical record or according to the observation of the researcher / assistant. They are traumatic lesions of the vulva, vagina and perineum in the absence of episiotomy.

5.9.4.2. Dependent Variables

• Duration of the third period of delivery - a continuous numerical variable (which can be later categorized), expressed in minutes, indicating the time of delivery of the placenta (started after the neonate exits), as registered by the researcher / assistant.

• Volume of blood loss one hour postpartum - a continuous numerical variable (which can be later categorized), expressed in milliliters (ml), indicating the amount of blood volume lost in the first hour postpartum. It will be evaluated by the collection of blood drained in the third period in a plastic bag designed for this purpose that will later be weighed and by weighing the compresses used in that period. - Weighing of plastic bags: quantified by the operator according to the weighing of plastic bags with drained blood in the third period of delivery. The weight in grams will be converted to milliliters by dividing the value in grams by 1.05 (blood density in grams per cubic centimeters). Continuous numerical variable expressed in milliliters (ml). - If a compress and / or gauze is used, the volume obtained will be added to the volume obtained by weighing the plastic bags. Each pack of 05 pack weighs 12 grams and each pack of gauze with 05 units weighs

08 grams. The compresses and gasses used shall be inserted in the following formula: N ° gauze pack used × 08 + N ° packet × 12 - total weight of gauzes and compresses at the end of delivery The value obtained in grams will be converted to milliliters by dividing the value in grams by 1.05 (blood density in grams per cubic centimeters) 25.

• Blood loss measured by the hematocrit difference - continuous numerical variable expressed as a percentage (%), referring to the punctual difference between the hematocrit before and after delivery (measured 24 hours postpartum) for each patient. The hematocrit before and after delivery will be measured according to the method of Wintrobe50 which consists of the dosage of the solid part of the blood (total mass of cells) in relation to the liquid part (plasma), measured in percentage points. Then it will be determined between two measures and will be used the calculation to estimate blood loss:

$$PSA = Volemia \times \frac{(Hi - Ht)}{\text{Mean hematocrit (initial and final)}}$$

at where:

• PSA = blood loss assessed (ml)

• Volemia = constant of the equation, considered as 6000 ml

• Hi = hematocrit before delivery

• Ht = hematocrit 24-48 h postpartum

• Postpartum haemorrhage (> 500 ml) in the first hour - dichotomous categorical variable, yes / no type. Expresses the presence or not of postpartum haemorrhage greater than 500 ml. Blood loss measured by weighing the plastic bags, weighing the compresses / gauzes and the hematocrit difference by the researcher / assistant.

• Severe postpartum haemorrhage (> 1000ml) in the first hour - dichotomous categorical variable, yes / no type. Expresses the presence or not of postpartum haemorrhage greater than 1000 ml. Blood loss measured by weighing the plastic bags, weighing the compresses / gauzes and the hematocrit difference by the researcher / assistant.

• Postpartum maternal hemoglobin less than 8 g / dl - dichotomous categorical variable, yes / no type, expressed the presence or absence of maternal hemoglobin 24-48 hours after delivery less than 8 g / dl. Collected 24-48 hours after delivery and the result will be later released in the institution's system and rescued by the researcher.

• Need for blood transfusion - dichotomous categorical variable, yes / no type. Defined as the accomplishment or not of the blood transfusion after the birth, according to observation of the researcher / assistant.

• Abdominal pain in the puerperium - dichotomous categorical variable, of the yes / no type, corresponding to the presence or absence of abdominal pain, according to the patient's information. • Need for therapeutic use of oxytocites in the first hour postpartum - dichotomous, dichotomic variable, yes / no type, defined as the need for oxytocin use in the third period, except for the two oxytocin ampules routinely used in all patients in the service during this period. Collected in medical records or as investigator / assistant.

• Need for therapeutic use of oxytocites within 24 h postpartum - dichotomous, dichotomic, yes / no variable, defined as the need to use complementary oxytocin in the third period within 24 h postpartum, except for the two oxytocin ampoules routinely used in all patients seen in the service during this period. Collected in medical records or as investigator / assistant. • Third period greater than 30 minutes - dichotomous categorical variable, yes or

no type, describing whether the time of placenta exit was over 30 minutes, according to the researcher / assistant.

• Third period greater than 60 minutes - dichotomous categorical variable, yes or no type, describing whether the time of placenta exit was greater than 60 minutes, according to observation of the researcher / assistant.

• Need for uterine healing - dichotomous categorical variable, yes / no type, expressing uterine healing, according to observation of the researcher / assistant. Uterine healing consists of revision of the uterine cavity or removal of placental debris using the hands.

- Manual removal of the placenta - dichotomous categorical variable, yes / no type, expressing whether or not the placenta is removed manually, according to the researcher / assistant.
- Need for uterine curettage - dichotomous categorical variable, yes / no type, expressing whether or not uterine curettage is performed, as observed by the researcher / assistant and / or records found in the medical record. Uterine curettage consists of the removal of placental remnants from the uterine cavity using instruments.
- Symptoms of anemia up to 48 h postpartum (dizziness, headache, fatigue) - dichotomous categorical variable, expressing the presence or absence of anemia symptoms up to 48 h postpartum. As noted by the researcher / assistant.
- Maternal satisfaction - a categorical polymorphic variable, defining whether the woman is satisfied with the management of the third period. It can vary between, very satisfied, satisfied, not very satisfied, not satisfied, very dissatisfied, according to the scale of faces (See item 5.10.6). According to observation or research of the researcher / assistant.

- 5.10. Procedures, tests, techniques and exams

5.10.1. Procedure for the Experimental Group It consists of the unclamping of the umbilical cord, which during the delivery was fixed to separate it with the placenta, where it will allow the blood of the placenta to drain freely into an appropriate container, 24 different from that used to measure the volume lost.

5.10.2. Procedures for the control group The cord will remain clamped after its clamping, until the placenta detaches.

5.10.3. Assessment of the duration of the third delivery period To evaluate the duration of the third period of childbirth will be used the clock that exists in each of the apartments in the sector. In both groups this evaluation will be initiated from the birth of the neonate to the exit of the placenta.

5.10.4. Assessment of blood loss in the first hour postpartum - Weigh the plastic bags: blood volume will be calculated by draining the blood that will be collected in a plastic bag designed for this purpose, where the researcher will place it under the buttocks of the puerperal immediately after delivery and will extend into a container stainless steel allowing the drainage of blood until the placenta leaves. Subsequently, this material will be weighed in an electronic scale where the weight will be obtained in grams and from there the volume will be obtained through the following formula:

d = Mass

Volume

at where:

• d = blood density 1.05 g / cm3 (grams per cubic centimeter) 25

• Mass = expressed in g (grams)

• Volume = expressed in cubic centimeters that will be converted to ml (1ml = 1cm3)

- If a compress and / or gauze is used, the volume obtained will be added to the volume obtained by weighing the plastic bags. Each pack of five pack weighs 12 grams and each pack of five gauze weighs 08 grams. The compresses and gasses used shall be inserted in the following formula:

N ° gauze pack used × 08 + N ° packet × 12 - total weight of gauzes and compresses at the end of delivery

The value obtained in grams will be converted to milliliters by dividing the value in grams by 1.05 (blood density in grams per cubic centimeters)

- Hematocrit difference: before delivery and 24-48 hours after delivery, the collection of 3 ml of puerperal blood will be performed to evaluate the hematocrit level in the blood. This material will be sent to the laboratory, where later the result of the exam will be released in the system of the institution and rescued by the researchers, which leaves on average with two hours. The cost of the exam will be the responsibility of the researchers if it is not approved by the Research Support Foundation / FAPE. If the result of the examination changes significantly, the woman will receive the care according to the protocol of the institution. This pre and postpartum hematocrit will be measured according to the method of Wintrobe50 which consists of the dosage of the solid part of the blood (total mass of cells) in relation to the liquid part (plasma), measured in percentage points. The difference between these two measures will then be determined and the calculation for estimating blood loss will be used:

$$PSA = \frac{Volemia \times (Hi - Ht)}{\text{Mean hematocrits}}$$

- where: PSA = blood loss assessed
- Volemia = constant of the equation, considered as 6000 ml
- Hi = hematocrit before delivery
- Ht = hematocrit 24-48 h postpartum

5.10.5. Assessment of hemoglobin levels 24-48 hours postpartum

After 24 hours work will be performed a 3ml collection of blood from the puerpera to evaluate the level of blood hemoglobin. This material will be sent to the laboratory, where the result of the pre-doctor will be, without research system and rescued by the researchers, which leaves on average with two hours. The case of success of the research of cases that were not attended by the Foundation of Research Support / FAPE The case of the program of research of cases of children who had a positive response to the process of selection of data.

5.10.6. Assessment of maternal satisfaction

The patient will be questioned, 24-48 h postpartum, by the researcher on the level of his satisfaction with the treatment that was applied to him. The satisfaction scale will be an association between the face scale (FIGURE 2) and the numerical scale. Each level will be clearly explained to the patient as follows:

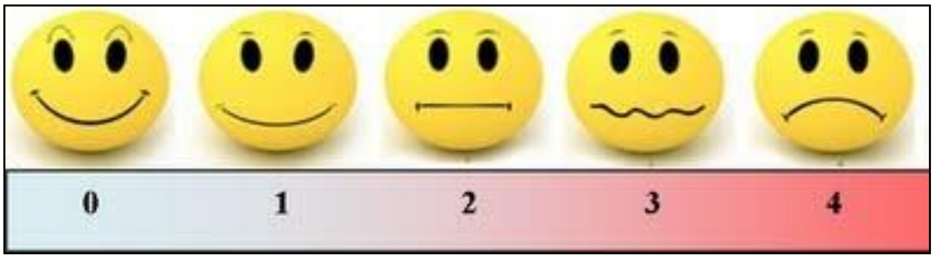

Figure 2: Face scale for evaluation of maternal satisfaction with the management of the third period • 0 - Very satisfied. • 1 - Satisfied. • 2 - Not satisfied. • 3 - Unsatisfied. • 4 - Very dissatisfied. For the purposes of analysis, the satisfaction will be recoded in satisfied (yes or no), corresponding yes to categories 0 and 1 above characterized51.

5.11. Criteria for discontinuation of the study

The study will be interrupted if secondary intercurrences from the method that causes any physical, moral or psychological harm to the patients are identified (extremely unlikely because they have never been described).

In the case of one group showing obvious differences in relation to the other and these differences make obvious the damage of drainage, or not drainage before the end of the trial, for ethical reasons, this will be closed and its results disclosed.

In addition, it should be emphasized that the patient's well-being will also be respected and her willingness to leave the study at any stage will be sovereign and unquestionable.

5.11.1. External Monitoring Committee (CME) The data monitoring committee will be a group of independent and external investigators who will ensure the progression and

safety of the data and, if necessary, criticize the effectiveness and may terminate the clinical trial. Only the CME may have information on clinical blinding when necessary.

> WEC members and their qualifications • Maria Inês Bezerra de Melo. IMIP obstetrical nurse. PhD student in Maternal and Child Health at IMIP. • Luciana Marques Andreto: Obstetrician Nurse of the Pernambuco Health College (FPS). PhD in Nutrition from UFPE. • José Natal Figueroa: IMIP postgraduate statistician. Member of the IMIP Research Ethics Committee. Post-graduate (PhD) in Maternal and Child Health by IMIP. • Carlos Noronha Neto: Gynecologist and Obstetrician at IMIP. Master's degree in Tocoginecology at the Amaury de Medeiros Health Center (CISAM). • Aurélio Costa. Gynecologist and Obstetrician from IMIP. PhD in Maternal and Child Health at IMIP.

Responsibilities of CME Members

• Luciana Marques Andreto: Group coordinator. Responsible for the appointment of the group's semi-annual meetings and to coordinate the discussions of the meetings.

• Carlos Noronha Neto: Secretary. Responsible for recording in minutes the discussions of the meetings and their decisions. In addition to requesting the collection forms and / or database to the researcher in charge.

• José Natal Figueroa: Statistics and Ethical Aspects. Responsible for interim analysis when pertinent and the ethical aspects involved in the study.

• Maria Inês Bezerra de Melo: Evaluator. Responsible for group discussions and final report.

• Aurélio Costa: Evaluator. Responsible for group discussions and final report.

Conflicts of Interest of WEC Members

• Maria Inês Bezerra de Melos: None.

• Luciana Marques Andreto: None.

• Carlos Noronha Neto: None.

• José Natal Figueroa: At the end of the study, the same can guide in the statistical analyzes of the study, if necessary.

• Aurélio Costa: None.

Frequency and format of WEC meetings

The meetings will be held semester in a reserved room at IMIP's postgraduate level, without the knowledge of the researchers directly involved with the study.

Initially an analysis of the frequency of the main outcomes will be performed, comparing with the frequency of these outcomes found in a systematic review carried out in IMIP and made available to the members of the CME. This analysis will be performed without breaking the confidentiality and without identifying the treatment group. If the frequency of unfavorable events is higher than expected for the sample studied, or if unexpected events occur, the CME should suggest that the interim analysis be performed.

Interim analysis plan

Interim analysis will be performed if some of the outcomes present a higher than expected frequency for the sample studied, and the confidentiality should be broken to see if one of the groups is causing greater risks to the participants and / or their concepts.

5.12. Data collection

5.12.1. Instrument for Data Collection

For data collection, a standard, pre-coded form for computer data entry (Appendix 3) will be used.

5.12.2. Procedures for collection

After the identification of the pregnant women that agree with the eligibility criteria and accept to participate in the research, having signed the TCLE, information will be collected and filled in the form. The checklist, as well as the form, will be completed by the researchers.

After completion, the forms will be rigorously reviewed by the researchers for checking the information collected with the information in the medical records. The elapsed time for data collection, adequate completion of forms and their revision should correspond to the schedule.

5.12.3 Control of information quality

The forms will be checked regularly and if inconsistencies are noticed, these will be analyzed by reviewing medical records or questioning the woman, if still hospitalized.

5.13. Data processing and analysis

5.13.1. Data Processing

The data will be typed into a specific database, created in the Epi-Info 3.7 statistical program. The data will be typed after review of the forms, in blocks of ten. Every month this database will be reviewed by the main researcher, obtaining a listing of the variables and correcting any inconsistencies or lack of information from the query to the forms.

It will be done double typing, in times and by different people (the principal investigator and an assistant). After typing, the databases will be compared. Consistency tests and

frequency distribution tables of the main variables will be obtained to correct any errors. In case of finding inconsistencies or lack of information, the forms will be consulted.

At the end of the typing, lists will be obtained again, for the final correction and creation of the definitive database, which will be subjected to the tests of cleaning and consistency of the information and from there will be performed the statistical analysis.

5.13.2. Data analysis

Data analysis will be performed by a blind "statistician" collaborator of the trial, using the statistical program Epi-Info 3.7. The analysis will be by intention to treat and will be performed with the groups identified as A or B and only at the end of this one, already prepared the tables, is that the one will have knowledge about which group corresponds to each letter.

To evaluate the association between the independent variable or predictor (use of placental drainage or not) and dependent variables (outcomes), double entry tables will be constructed. For the continuous numerical variables of normal distribution, Student's t-test will be used, if the normal distribution is not verified (Kolmogorov-Smirnov test), the Mann-Whiney test will be used. For categorical variables, Chi-square association tests (Pearson) and Fisher's exact test, where relevant (one of the expected values less than five) will be used. All adopted p values will be counted. The Risk Ratio (RR) will be calculated as a relative risk measure, as well as the 95% Confidence Interval (95% CI). The default value of 1.0 will be assigned to the reference category. The Number Needed to Treat and Obtain a Benefit (NNT) and Number Required to Treat and Achieve Harm (NNH), with their respective 95% confidence intervals, shall be calculated for outcomes that demonstrate a statistically significant association with drainage placenta. This calculation will be done using the EBM Calculator public domain program.

5.14. Ethical aspects

The present research responds to the postulates of the Declaration of Helsinki amended in Seoul 2009, and follows the terms recommended by the National Health Council (Resolution 196 of 1996) for research on human beings. The project will be submitted to the Research Ethics Committee (CEP) of IMIP, after being authorized by the medical coordination of CAM-IMIP and Maternidade Petronila Campos.

All patients will be informed about the objectives and methods of the study, and only those who agree to participate will be included, by signing the Informed Consent Form (Appendix 2). It is clearly safeguarded the right of any patient to refuse to participate in the study, being guaranteed the guarantee of treatment for all, regardless of their participation.

The research will begin only after approval by the Research Ethics Committee of IMIP and will be interrupted in the presence of serious side effects or if demonstrated in the interim analysis of the superiority of one of the treatments.

It is emphasized that the use of placental drainage is not a standard procedure in the services involved, since the professionals who attend the puerpera do not follow a standardized approach regarding the alternatives used to reduce the time of the third period and the blood loss in this period. In this way, some professionals indicate the use of placental drainage, because they believe particularly in its effects, and others do not.

According to the results of the present study, the routine of the institution could be changed, establishing a protocol to use or not the placental drainage in puerperal women.

The researchers commit to publish the results of the study in indexed journals, regardless of whether they favor placental drainage.

5.14.1. Consent Form Free and Informed

Before being included as a research sample, the woman will be clarified by the researcher about the objectives, justification and methodology of the clinical trial with accessible and easily understood language for the same.

Once all doubts have been clarified, the possible participant will be invited to sign the TCLE (Appendix 2), which will consist of all the items provided for in Resolution 196/96 of the National Health Council. This Term will be read aloud to each candidate to participate in the study, clarifying any doubts during its reading.

5.14.2. Conflict of interests

This research is free of conflicts of private or institutional interest. The equipment for the research will be acquired with resources of the researchers themselves.
